# Detection of protein, starch, oil, and moisture content of corn kernels using one-dimensional convolutional autoencoder and near-infrared spectroscopy

**DOI:** 10.7717/peerj-cs.1266

**Published:** 2023-03-09

**Authors:** Ozcan Cataltas, Kemal Tutuncu

**Affiliations:** Faculty of Technology, Selcuk University, Konya, Turkey

**Keywords:** Near-infrared spectroscopy, Chemometrics, Cereal analysis, Convolutional autoencoder, Multiple linear regression

## Abstract

**Background:**

Analysis of the nutritional values and chemical composition of grain products plays an essential role in determining the quality of the products. Near-infrared spectroscopy has attracted the attention of researchers in recent years due to its advantages in the analysis process. However, preprocessing and regression models in near-infrared spectroscopy are usually determined by trial and error. Combining newly popular deep learning algorithms with near-infrared spectroscopy has brought a new perspective to this area.

**Methods:**

This article presents a new method that combines a one-dimensional convolutional autoencoder with near-infrared spectroscopy to analyze the protein, moisture, oil, and starch content of corn kernels. First, a one-dimensional convolutional autoencoder model was created for three different spectra in the corn dataset. Thirty-two latent variables were obtained for each spectrum, which is a low-dimensional spectrum representation. Multiple linear regression models were built for each target using the latent variables of obtained autoencoder models.

**Results:**

R^2^, RMSE, and RMSPE were used to show the performance of the proposed model. The created one-dimensional convolutional autoencoder model achieved a high reconstruction rate with a mean RMSPE value of 1.90% and 2.27% for calibration and prediction sets, respectively. This way, a spectrum with 700 features was converted to only 32 features. The created MLR models which use these features as input were compared to partial least squares regression and principal component regression combined with various preprocessing methods. Experimental results indicate that the proposed method has superior performance, especially in MP5 and MP6 datasets.

## Introduction

Near-infrared spectroscopy (NIRS) has become a widely used method in recent years due to its fast and low-cost analysis capability and non-destructive feature (Roggo et al., 2007; Yi et al., 2017; Zhang et al., 2019). Although it is used in many different fields, the main application of NIRS is the food industry (Rajput et al., 2017). According to Chen, Lin & Zhao (2021), NIRS is the most studied method for the non-destructive analysis of food products. It is widely used to determine the quality parameters of grain products (Chen, Tan & Lin, 2018; Cheng, Vella & Stasiewicz, 2019), to determine the freshness of fruits and vegetables (Huang, Lu & Chen, 2018; Mishra et al., 2021; Yuan et al., 2020; Zhu & Tian, 2018), detection of insects in food products (Johnson, 2020; Santos et al., 2019), detection of meat and chicken fraud (Krepper et al., 2018; López-Maestresalas et al., 2019; Mabood et al., 2020), detection of adulteration on expensive products (De Girolamo et al., 2020; Genis, Durna & Boyaci, 2021; Laborde et al., 2021; Rodionova et al., 2021; Yang et al., 2017), analysis of dairy products (Mabood et al., 2017; Mohamed et al., 2021; Pereira et al., 2020; Yang et al., 2020), and the analysis of beverages (Genisheva et al., 2018).

Near-infrared (NIR) spectra are obtained from portable, stationary, or in-line spectrometers. These spectrometers essentially comprise one or more light sources, a sensor, and other optical elements. Therefore, the NIR spectrum needs to be preprocessed as it contains sensor, light, or converter-induced distortions (Mishra et al., 2020). Although dozens of methods are used in the literature, the most commonly used preprocessing methods can be said as mean scatter correction (MSC), standard normal variate (SNV), Savitzky-Golay filter (SG), first and second derivative and mean centering (MC) (Çataltaş & Tütüncü, 2021; Rinnan, Berg & Engelsen, 2009). However, the preprocessing method is determined by trial and error since the usefulness of the preprocessing method varies according to each spectrum. Therefore, choosing the most appropriate preprocessing method is one of the crucial steps of NIRS and directly affects the accuracy of the system. Although some efforts have been made to solve the preprocessing problem, satisfactory results have not been reached yet (Helin et al., 2022).

In order to obtain meaningful information from the new spectrum obtained after preprocessing, quantitative and qualitative analyzes are carried out using various regression and machine learning methods. As quantitative analysis methods, partial least squares regression (PLSR), principal component regression (PCR), and multiple linear regression (MLR) methods are generally used, while partial least squares discriminant analysis (PLS-DA) and linear discriminant analysis (LDA) are used for qualitative analysis (Popovic et al., 2019; Roggo et al., 2007; Teye et al., 2020). These methods, also called chemometrics, are used because they offer ease of application. This feature provides convenience, especially in in-line systems with low-capacity processors. However, most calibration methods with successful results in the literature are not user-friendly in laboratory systems as they contain preprocessing methods which are special for spectra (Chen, Lin & Zhao, 2021). On the other hand, chemometric methods cannot successfully reveal non-linear relationships because they create a linear model between input and output variables. For this purpose, kernel-based methods such as SVM and machine learning methods such as artificial neural networks (ANN) and deep learning (DL) are increasingly used in NIRS.

The widespread use of machine learning algorithms has brought a different perspective to spectrum data processing, as in all other fields. In particular, the extensive use of computers with increasing computing power has intensified studies on ANNs and deep learning methods (Chen et al., 2020). Deep learning is a popular method based on ANN and can extract high-level features using stacked network layers. Due to this feature, it has an increasing use in the field of spectroscopy for noise reduction, feature extraction, classification, and regression (Yang et al., 2022b). Acquarelli et al. (2017), Cui & Fearn (2018), Kim et al. (2023) and Malek, Melgani & Bazi (2018) are some example studies that used CNN for quantitative or qualitative analysis. Chemometrics has generalization problems when used on a new instrument. Calibration transfer between different instruments is a popular application of deep learning on NIRS to address this problem. Yang et al. (2022a) has developed a deep learning model with three stacked convolutional layers for calibration transfer. They used five instruments and two datasets (soybean and wheat) to validate model performance. The comparison with the conventional standardization method showed that critical features could be protected during calibration transfer between different instruments. While they obtained comparable RMSE values with the soybean dataset using CNN and PLSR (0.078 and 0.076), in the wheat dataset, CNN outperformed the PLSR method (0.053 and 0.130). Mishra & Passos (2021) conducted a similar study with the tablet dataset and olive dataset. They used two instruments: one for primary model development and the other for calibration transfer. Fine-tuning was performed on fully connected and convolutional layers of the model while protecting convolutional layers. The RMSE value of 3.258, which is lower than that obtained with instrument 1 (3.513), was obtained with instrument 2 using the calibration transfer method. Another deep learning model was developed by Yang et al. (2022b) to reduce the impact of interseasonal variations on spectral analysis. They used Cuiguan pear, Rohca pear and mango datasets to validate the calibration transfer model and obtained RMSE values for each dataset that were at least 9.2%, 17.5%, and 11.6% lower than conventional methods. These studies have presented promising results for device dependency, which is a critical problem in NIRS.

Autoencoders, a special type of deep learning, aim to obtain a valuable representation of the input data while providing an output precisely like the given input data. Because of this property, autoencoders are classified as unsupervised learning methods. Autoencoders are often used for feature extraction, noise removal, or outliner detection. Stacked autoencoder, sparse autoencoder, convolutional autoencoder, and variational autoencoder are commonly used types of autoencoders (An et al., 2022). Le (2020) has proposed a model combining a stacked sparse autoencoder with affine transform—extreme learning machine to detect the amylose content of rice and the moisture content of corn. He obtained the correlation coefficient value in the prediction set of 0.999 for the moisture parameter and 0.927 for the amylose parameter, meaning that this model showed better performance than the partial least squares regression and the extreme learning machine. Mu & Chen (2022) have proposed a variational autoencoder-based transfer model to deal with unlabeled spectrum problems in practical NIRS applications. They performed the test study using the dataset they created in the simulation environment. They achieved a high R^2^ value of 0.9988 with their proposed autoencoder model, outperforming nine different methods compared. Another application of the autoencoder was performed by Said, Wahba & Khalil (2022) to analyze the fat content of cow milk and detect water adulteration. They obtained R^2^ values between 0.914 and 0.966 for fat content prediction and 0.411–0.910 for water adulteration detection.

This study combines a one-dimensional convolutional autoencoder (1D-CAE) with NIRS to provide a convenient method for qualitative analysis. First, unlike the other studies, feature extraction was performed using a 1D-CAE model from the spectral data of corn kernels. Then, the obtained features were utilized in MLR modeling to determine the moisture, oil, protein, and starch parameters of corn kernels. The proposed method was tested on three different spectra of corn kernels obtained from three devices. The proposed method was compared with conventional chemometric methods and the literature.

## Materials & Methods

### Dataset description

**Figure 1:**
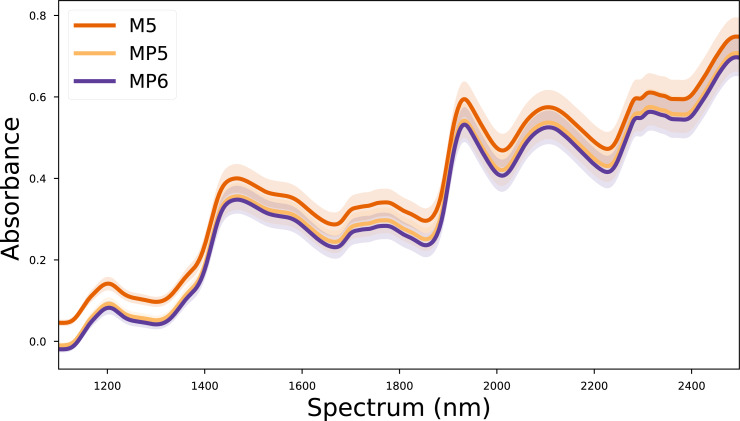
Mean spectra of the corn dataset for each device.

In this study, the corn dataset, which is commonly used in the literature, was used to test the validity of the proposed method. The corn dataset contains the spectra of 80 corn kernels measured on three different devices. These devices are called M5 (FOSS NIRSystems 5000), MP5 (FOSS NIRSystems 5000), and MP6 (FOSS NIRSystems 6000). The wavelength range covered in the dataset is 1,100–2,498 nm at 2 nm intervals. In addition, the reference values for moisture, oil, protein, and starch targets of each corn kernel are also included in the dataset. The mean spectra for each device in the corn dataset are given in Fig. 1. The corn dataset can be accessed from (https://eigenvector.com/resources/data-sets/).

### Autoencoders

**Figure 2:**
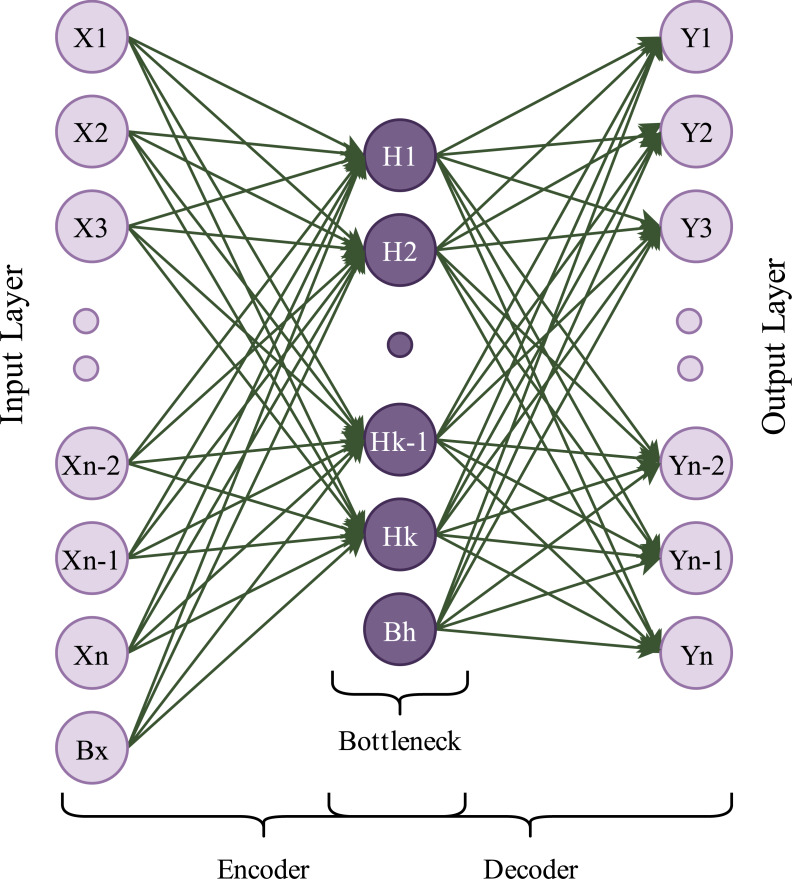
A simple autoencoder model.

Autoencoders are generative and unsupervised neural network algorithms. In this learning algorithm, the main goal is to get output values equal to input values. An autoencoder framework consists of two main blocks: encoder and decoder. The encoder block compresses input data into a low-dimensional representation called latent variables, which contains valuable input data information. The decoder block takes these latent variables as input and attempts to obtain the original data. An autoencoder framework also includes a waypoint, called bottleneck, between the encoder and the decoder (Zhang, Liu & Jin, 2020a). A simple diagram of an autoencoder is shown in Fig. 2.

For a given input data *x*_*i*_, *i* = 1, 2, …, *N*, latent variables, *h*_*i*_, can be obtained as:

(1) \begin{eqnarray*}{h}_{i}=\psi \left( {w}_{i}\ast {x}_{i}+b \right) \end{eqnarray*}

where *w*_*i*_ denotes coefficients, *b* denotes biases and $\psi \left( x \right) $ denotes the activation function of the encoder layer. After the encoding process, the decoding process starts with obtained *h*_*i*_ using (2):

(2) \begin{eqnarray*}{y}_{i}=\psi \left( {\tilde {w}}_{i}\ast {h}_{i}+\tilde {b} \right) \end{eqnarray*}

here, ${\tilde {w}}_{i}$ and $\tilde {b}$ denote coefficients and biases of the decoder layer. In an ideal autoencoder, *x*_*i*_ and *y*_*i*_ are expected to be equal. During the training phase, the created network model tries to minimize the loss function, $J \left( \theta \right) $, by searching for optimal values for the weight and bias parameters.

(3) \begin{eqnarray*}J \left( \theta \right) = \frac{1}{N} \sum _{i=1}^{N}({x}_{i}-{y}_{i})^{2}.\end{eqnarray*}

### 1D convolutional autoencoders

Convolutional neural network (CNN) is a special form of neural networks that uses convolution operation in layers. Previous works show that convolutional layers are more successful than fully connected layers in retrieving high-level features (Kiranyaz et al., 2021). Because of this, CNN has taken the field of artificial intelligence to an advanced level by adding a different depth. Similarly, a convolution operation can be applied to layers of the autoencoder network. Thus, a convolutional autoencoder can extract high-level features that can be used for classification or regression. CNNs are mainly used for high-dimensional data such as images; however, they can also be applied to low-dimensional data such as signals or time series.

The mathematical convolution operation of two discrete signals in one dimension can be defined as follows:

(4) \begin{eqnarray*}y[n]=x[n]\ast h[n]=\sum _{k=-\infty }^{\infty }x \left[ k \right] h \left[ n-k \right] .\end{eqnarray*}

Where $x \left( i \right) $, $y \left( i \right) $ and $h \left( i \right) $ are input, output, and filter vectors, respectively.

For a given vector, *x*, and 1D filter, *w*, whose length is *m*, the convolution formula can be reorganized as:

(5) \begin{eqnarray*}\mathrm{conv}(x,w)_{k}=\sum _{i=1}^{m}{w}_{i}.{x}_{k+i-1}.\end{eqnarray*}

In the forward propagation step of the 1D CNN network, we can generalize formula (5) for each neuron in each CNN layer.

(6) \begin{eqnarray*}{x}_{k}^{l}=\psi \left( \mathrm{conv} \left( {x}_{k}^{l-1},{w}_{k}^{l-1} \right) +{b}_{k}^{l} \right) =\psi \left( \sum _{i=1}^{m}{w}_{k}^{l-1}.{x}_{k+i-1}^{l-1}+{b}_{k}^{l} \right) .\end{eqnarray*}

Here, ${b}_{k}^{l}$ is the bias of the *k* th neuron at layer *l*, ${x}_{k}^{l-1}$ is the output of layer *l* − 1, ${w}_{k}^{l-1}$ is filter coefficients of the *k* th neuron at layer *l* − 1, *ψ*(*x*) is the activation function of the current layer. An activation function is used to ensure the nonlinearity of the system. The selection of the activation function is one of the essential phases in creating a network model. Sigmoid, hyperbolic tangent (tanh), and rectified linear activation function (ReLU) are the most popular activation functions. Of these, the hyperbolic tangent is preferred when the input and output are constrained to values between −1 and 1 (Patil & Kumar, 2021). The formula of the hyperbolic tangent activation function is given in Eq. (7).

(7) \begin{eqnarray*}\psi \left( x \right) = \frac{{e}^{x}-{e}^{-x}}{{e}^{x}+{e}^{-x}} .\end{eqnarray*}

**Figure 3:**
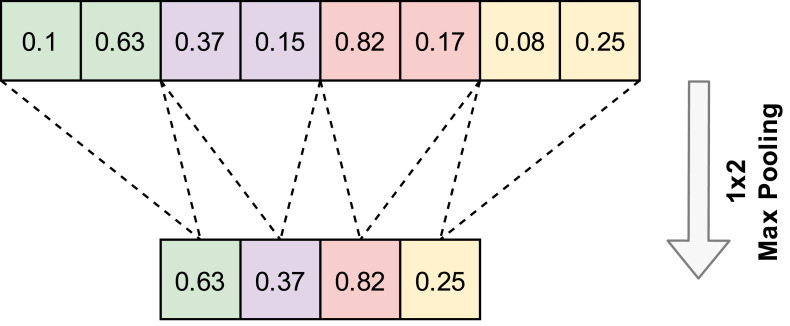
An example application of one-dimensional max pooling.

A convolution layer is generally followed by a pooling layer. The pooling layer reduces features without changing the number of channels. Pooling layers do not contain any parameters, so no learning occurs in this layer. Max pooling and average pooling are the most popular pooling algorithms. An example of the 1D-Max pooling used in this study is given in Fig. 3.

A loss function needs to be utilized to evaluate the learning progress of the network. Mean squared error is the most preferred loss function for regression tasks, and its formula is given in Eq. (8).

(8) \begin{eqnarray*}J= \frac{1}{N} \sum _{i=1}^{N}{ \left( {\hat {y}}_{i}-{y}_{i} \right) }^{2}.\end{eqnarray*}

Here, ${\hat {y}}_{i}$ is the predicted output, and *y*_*i*_ is the target output of the network, which is also equal to the input, *x*_*i*_. For backward propagation, gradients must be calculated and propagated from the output layer to the input layer using the chain rule.

(9) \begin{eqnarray*} \frac{\partial J}{\partial {w}_{k}} =\sum _{i=1}^{N-m+1} \frac{\partial J}{\partial {x}_{i}^{l}} . \frac{\partial {x}_{i}^{l}}{\partial {w}_{k}} .\end{eqnarray*}

In Eq. (9), $ \frac{\partial {x}_{i}^{l}}{\partial {w}_{k}} $ term can be calculated as in Eqs. (10) and (11)

(10) \begin{eqnarray*} \frac{\partial {x}_{i}^{l}}{\partial {w}_{k}^{l}} & = \frac{\partial \left( \psi \left( \sum _{i=1}^{m}{w}_{k}^{l-1}.{x}_{k+i-1}^{l-1}+{b}_{k}^{l} \right) \right) }{\partial {w}_{k}^{l}} \end{eqnarray*}

(11) \begin{eqnarray*} \frac{\partial {x}_{i}^{l}}{\partial {w}_{k}^{l}} & ={\psi }^{{}^{{^{\prime}}}}({x}_{k+i-1}^{l-1}).\end{eqnarray*}

Substituting Eqs. (11) into (9), the gradients needed to update the weights are obtained as in Eq. (12).

(12) \begin{eqnarray*} \frac{\partial J}{\partial {w}_{k}} =\sum _{i=1}^{N-m+1} \frac{\partial J}{\partial {x}_{i}^{l}} .{\psi }^{{}^{{^{\prime}}}} \left( {x}_{k+i-1}^{l-1} \right) .\end{eqnarray*}

Various optimization algorithms in the literature have been proposed to update weights and biases, such as Stochastic Gradient Descent (SGD), RMSProp, Adam, and Adadelta. Among these, the Adam optimizer was used in our study. The Adam optimizer is based on adaptive moment estimation and combines Momentum and RMSProp (Kingma & Ba, 2014). To apply the Adam optimizer, firstly, the moving averages of the gradients and the moving averages of the square gradients, *m*^*t*^ and *v*^*t*^, needs to be calculated using formulas Eqs. (13) and (14).

(13) \begin{eqnarray*}{m}^{t}& ={\beta }_{1}{m}^{t-1}+(1-{\beta }_{1}) \frac{\partial J}{\partial {w}_{k}} \end{eqnarray*}

(14) \begin{eqnarray*}{v}^{t}& ={\beta }_{2}{v}^{t-1}+(1-{\beta }_{2}) \frac{\partial {J}^{2}}{\partial {w}_{k}^{2}} .\end{eqnarray*}

Where, *β*_1_ and *β*_2_ are decay rate parameters. Using Eqs. (13) and (14), we can calculate bias corrected *m*^*t*^ and *v*^*t*^.

(15) \begin{eqnarray*}{\hat {m}}^{t}& = \frac{{m}^{t}}{(1-{\beta }_{1}^{t})} \end{eqnarray*}

(16) \begin{eqnarray*}{\hat {v}}^{t}& = \frac{{v}^{t}}{(1-{\beta }_{2}^{t})} .\end{eqnarray*}

Using Eq. (17), we can update weights and biases.

(17) \begin{eqnarray*}{w}_{k}^{ \left( t \right) }={w}_{k}^{(t-1)}-\eta \frac{{\hat {m}}^{t}}{\sqrt{{\hat {v}}^{t}}+\in } .\end{eqnarray*}

Here, *η* is named as the learning rate, another critical hyperparameter affecting the learning speed of the network.

### Proposed model

**Figure 4:**
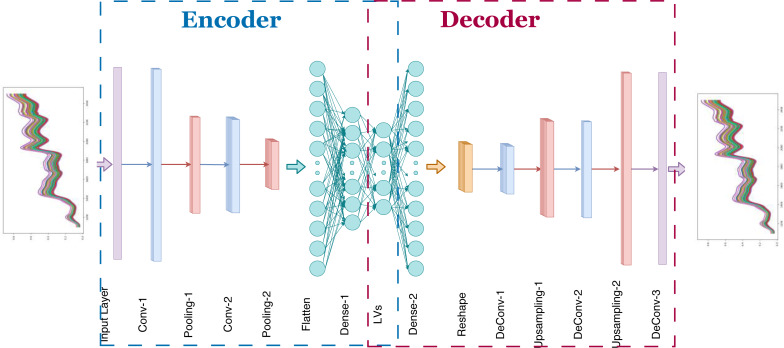
Proposed one dimensional convolutional autoencoder model.

In Fig. 4, the proposed 1D-CAE model is shown. This model consists of two convolutional layers, two pooling layers, and two dense (fully connected) layers in the encoder sub-model and three convolutional layers, two upsampling layers, and one dense layer in the decoder sub-model. The main reason for choosing two stacked convolutional layers in our model is that some previous works show that two or three layers are sufficient for CNN-based NIRS applications (Zhang et al., 2020b). Using random search, the optimal number of filters for each convolutional layer in the encoder and decoder model was determined and given in Tables 1 and 2. The hyperbolic tangent function was chosen to provide nonlinearity. The filter weights were randomly initialized. Training of the autoencoder model was done using randomly chosen samples. The reference values in the dataset were not used in this process which is unsupervised learning. The backpropagation algorithm was used to update the convolution filter weights. Although two, four, eight, 16, and 32 neurons were tried as the number of latent variables, no remarkable success was achieved in models containing fewer than 32 neurons, forcing us to select 32 neurons in our model. The optimization of unsupervised learning was done using the ADAM optimizer (Kingma & Ba, 2014). Selected values for learning rate, *β*_1_ and *β*_2_ were 0.001, 0.9, and 0.999, respectively. After the unsupervised learning training process, 32 latent variables for each corn kernel were exported for further analysis.

**Table 1:** Description of encoder sub-model of convolutional autoencoder architecture used in our study.

No	Type	Number of filters	Kernel/ Pool size	Stride	Output shape	Number of parameters
1	Input	–	–	–	(700,1)	0
2	Conv1D	16	(5)	1	(700, 16)	96
3	Max Pooling	–	(2)	0	(350, 16)	0
4	Conv1D	32	(5)	1	(350, 32)	2,592
5	Max Pooling	–	(2)	0	(175, 32)	0
6	Flatten	–	–	–	(5600)	0
7	Dense	–	–	–	(64)	358,464
8	Dense	–	–	–	(32)	2,080

**Table 2:** Description of decoder sub-model of convolutional autoencoder architecture used in our study.

No	Type	Number of filters	Kernel/ Pool size	Stride	Output shape	Number of parameters
1	Input	–	–	–	(32)	0
2	Dense	–	–	–	(5600)	184,800
3	Reshape	–	–	–	(175, 32)	0
4	Conv1D_Transpose	32	(3)	1	(175, 32)	3,104
5	Up-sampling	–	(2)	0	(350, 32)	0
6	Conv1D_Transpose	16	(3)	1	(350, 16)	1,552
7	Up-sampling	–	(2)	0	(700, 16)	0
8	Conv1D_Transpose	1	(3)	1	(700, 1)	49

In the second part of the proposed model, multiple linear regression was employed to establish linear relations between latent variables and reference outputs. For each reference output, moisture, oil, protein, and starch, different MLR models were developed using the same latent variables. In addition, all the processes mentioned above were performed for three devices in the corn dataset to confirm that our results are device independent.

### Dataset processing

In order to create a reliable model and to make accurate comparisons with known methods, the spectral data in the dataset were divided into two sets: one for calibration and one for prediction by the random division method. This way, the same samples from different devices were used for calibration and prediction. Of 80 samples, 60 were labeled as the calibration set, and the remaining 20 were labeled as the prediction set. While the calibration set was used to train the model, the prediction set was used to evaluate the model’s performance. The reason for splitting the dataset into two subsets is that for small datasets, the additional splitting can result in a smaller training set which can be subject to overfitting (Ashtiani et al., 2021; Féré et al., 2020). Statistics of the split dataset are given in Table 3.

**Table 3:** Statistics of the split dataset.

Target	Calibration			Prediction			Total		
	**Min**	**Mean**	**Std**	**Min**	**Mean**	**Std**	**Min**	**Mean**	**Std**
	**Max**			**Max**			**Max**		
Moisture	9.38	10.23	0.38	9.64	10.23	0.37	9.38	10.23	0.38
	10.99			10.94			10.99		
Oil	3.09	3.50	0.17	3.11	3.51	0.20	3.09	3.50	0.18
	3.82			3.83			3.83		
Protein	7.65	8.67	0.50	7.79	8.67	0.49	7.65	8.67	0.50
	9.69			9.71			9.71		
Starch	62.88	64.68	0.81	62.83	64.73	0.83	62.83	64.70	0.82
	66.47			65.81			66.47		

**Notes.**

All values in the table are in percent.

### Hyperparameters

In machine learning, tuning the hyperparameters of a model is an essential step that determines the performance of the model. In this work, the optimization of hyperparameters was carried out using random search with a lookup table. This table includes kernel size, the number of latent variables, and batch size. The lookup table is given in Table S1. During training, the maximum number of epochs was set to 20. Validation loss was tracked along the training of the model. When validation loss increased for two consecutive epochs, the training of the network was stopped to avoid overfitting.

### Test environment

In this article, autoencoder and regression models were implemented in Python (version 3.7.13) using Keras (version 2.9.0), which is a high-level neural networks library (Chollet, 2022) and scikit-learn (version 1.0.2) which provides regression models and model evaluation tools (Lemaitre, 2021). All training and testing processes were performed using a computer with Intel i7 10870H CPU, 16GB Ram, and Nvidia RTX 2070 GPU.

## Results

### Performance evaluation criteria

The coefficient of determination (R^2^), root mean squared error (RMSE), and root mean squared percentage error (RMSPE) indicators were used to test the evaluation of our proposed model. R^2^ and RMSE indicators were used for overall model evaluation, while RMSPE was used to determine the performance of the autoencoder model in reconstructing the input spectrum. The formulas of R^2^, RMSE, and RMSPE are given in Eqs. (18), (19) and (20) (Ashtiani, Salarikia & Golzarian, 2017; Chen et al., 2008; Miles, 2005).

(18) \begin{eqnarray*}{R}^{2}& =1- \frac{\sum _{i=1}^{N}({y}_{i}-{\hat {y}}_{i})^{2}}{\sum _{i=1}^{N}({y}_{i}-{\overline{y}}_{i})^{2}} \end{eqnarray*}

(19) \begin{eqnarray*}\mathrm{RMSE}& =\sqrt{ \frac{1}{N} \sum _{i=1}^{N}({y}_{i}-{\hat {y}}_{i})^{2}}\end{eqnarray*}

(20) \begin{eqnarray*}\mathrm{RMSPE}& =100\ast \sqrt{ \frac{1}{N} \sum _{i=1}^{N}{ \left( \frac{{y}_{i}-{\hat {y}}_{i}}{{y}_{i}} \right) }^{2}}.\end{eqnarray*}

Here *N* is the sample size, *y*_*i*_, ${\hat {y}}_{i}$ and ${\overline{y}}_{i}$ are the actual output, the predicted output, and the mean value of actual outputs, respectively. As one can understand from Eq. (18), *R*^2^ indicator is the proportion of the dependent variable variation explained by the independent variables and takes values between 0 and 1. RMSE, another indicator often used in regression tasks, is equal to the standard deviation of the residuals. Similarly, RMSPE gives the ratio of the error to the input spectrum in percent. Values closer to 0 are preferable for RMSE and RMSPE. Although most studies include the ratio of performance to deviation (RPD) metric to show model quality, some articles argue that RPD is not different from R^2^ (Minasny & McBratney, 2013). For this reason, we did not find it necessary to include both metrics.

**Figure 5:**
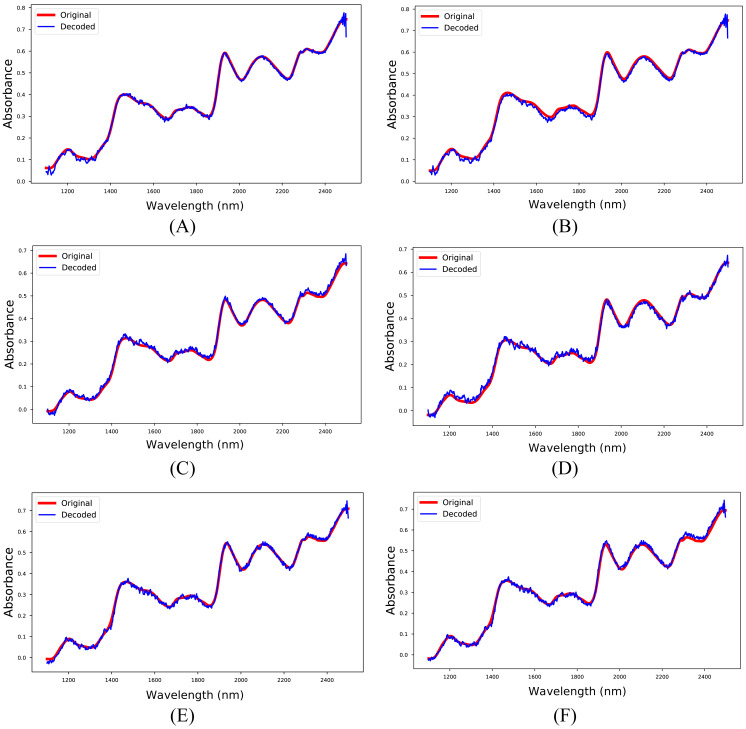
Input and reconstructed spectra. (A) M5-training, (B) M5-test, (C) MP5-training, (D) MP5-test, (E) MP6-training, (F) MP6-test.

The 1D-CAE model was created separately for M5, MP5, and MP6 datasets in the first experiment. In this stage, the main objective was to obtain a reliable model that reconstructs the spectrum like the input spectrum and to obtain meaningful latent variables. The RMSPE indicator was utilized to show the reconstruction performance of the model, and the obtained results are given in Table 4. Besides, sample input and reconstructed spectra for each dataset are shown in Fig. 5. The 1D-CAE model successfully reconstructed the spectrum and obtained a mean RMSPE value of 1.90% for calibration and 2.27% for prediction.

**Table 4:** RMSPE results between the input and decoded spectra using the 1D convolutional autoencoder model.

Dataset Model	M5		MP5		MP6	
	**Min**	**Mean**±**Std**	**Min**	**Mean**±**Std**	**Min**	**Mean**±**Std**
	**Max**		**Max**		**Max**	
Calibration	0.69	2.62 ± 1.84	0.35	1.00 ± 0.73	0.83	2.09 ± 1.13
	8.59		4.01		5.86	
Prediction	0.89	3.31 ± 2.30	0.43	0.98 ± 0.61	0.84	2.53 ± 1.27
	10.70		3.08		5.76	

**Notes.**

All values in the table are in percent.

**Figure 6:**
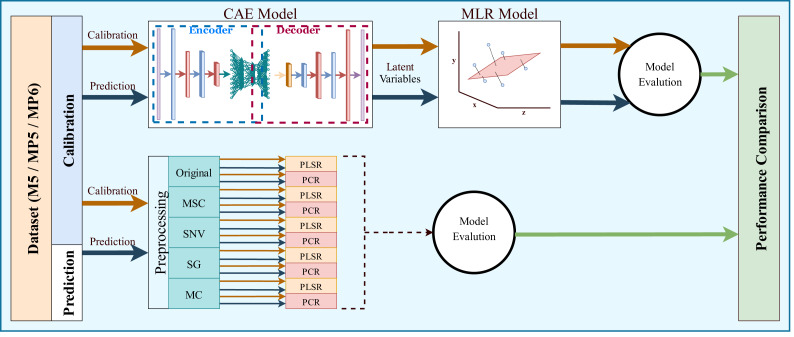
Overall block diagram of the application and comparison steps for the proposed method.

The most common regression methods used in NIR systems, PLSR and PCR, were used as comparison methods. Another popular regression model, MLR, was not used because the sample number is lower than the feature number, which is necessary for MLR models. The latent variable and principal component parameters of the PLSR and PCR methods were selected as the optimal value between 1 and 10. RMSE and 5-fold cross-validation were used to find the optimal value for the latent variable and principal component parameters. Together with the original spectrum, four different preprocessing methods were applied to spectral data to increase the accuracy of these methods. Besides, the proposed method, 1D-CAE+MLR, was also applied to spectrum data. R^2^ and RMSE values were calculated separately for each combination. The block diagram summarizing the whole process is given in Fig. 6.

The M5 dataset is the first used for the test, and the obtained results are given in Table 5. A careful examination of these results indicates that the proposed method exhibits satisfactory performance compared to other methods, as evidenced by the minimum R^2^ values of 0.9560 and 0.9012 in the calibration and prediction sets, respectively. Furthermore, the proposed method demonstrated superior performance when predicting oil and starch content, as it yielded higher R^2^ values and lower RMSE values. However, when analyzing the prediction of moisture and protein content, it was found that the PLSR method yielded higher R^2^ values and lower RMSE values.

**Table 5:** Obtained results for the M5 dataset.

Target	Preprocessing method	Regressor	LV	R^2^		RMSE	
			**PC**	**Calibration**	**Prediction**	**Calibration**	**Prediction**
Moisture	Original	PLSR	10	0.9978	**0.9957**	0.0175	0.0245
			MSC		6	0.8931	0.8104	0.1235	0.1645
			SNV		6	0.8916	0.7961	0.1244	0.1706
			SG		10	0.9978	**0.9957**	0.0175	**0.0245**
			MC	10	**0.9982**	0.9939	**0.0160**	0.0294
		Original	PCR	9	0.9903	0.9833	0.0370	0.0487
		MSC			8	0.8843	0.7741	0.1285	0.1796
		SNV			8	0.8813	0.7503	0.1301	0.1888
		SG			9	0.9903	0.983	0.0371	0.0487
		MC		8	0.9910	0.9865	0.0358	0.0438
	1D CAE + MLR			0.9913	0.9716	0.0353	0.0628
Oil	Original	PLSR	10	0.9254	0.7554	0.0480	0.0870
			MSC		8	0.8680	0.7256	0.0639	0.0921
			SNV		8	0.8674	0.7222	0.0640	0.0927
			SG		10	0.9248	0.7548	0.0482	0.0871
			MC	10	0.9373	0.8220	0.0440	0.0742
		Original	PCR	8	0.6856	0.4450	0.0986	0.1309
		MSC			9	0.7949	0.6579	0.0796	0.1028
		SNV			9	0.7931	0.6579	0.0800	0.1029
		SG			8	0.6855	0.4455	0.0986	0.1310
		MC		9	0.7970	0.6187	0.0792	0.1086
	1D CAE + MLR			**0.9733**	**0.9632**	**0.0272**	**0.0388**
Protein	Original	PLSR	10	0.9630	0.9172	0.0953	0.1425
			MSC		10	0.9657	0.9408	0.0917	0.1204
			SNV		10	0.9666	0.9410	0.0904	0.1203
			SG		10	0.9628	0.9170	0.0955	0.1427
			MC	10	0.9729	**0.9484**	**0.0815**	**0.1125**
		Original	PCR	9	0.8848	0.8038	0.1681	0.2194
		MSC			8	0.9350	0.9092	0.1262	0.1492
		SNV			8	0.9371	0.9118	0.1242	0.1471
		SG			9	0.8847	0.803	0.1681	0.2195
		MC		8	0.9092	0.8476	0.1492	0.1934
	1D CAE + MLR			**0.9731**	0.9012	0.0816	0.1535
Starch	Original	PLSR	10	0.9525	0.8538	0.1777	0.3117
			MSC		10	0.9517	0.8617	0.1792	0.3032
			SNV		10	0.9521	0.8615	0.1783	0.3034
			SG		10	0.9523	0.8539	0.1781	0.3116
			MC	10	**0.9728**	0.9304	**0.1344**	0.2150
		Original	PCR	8	0.7341	0.3903	0.4205	0.6367
		MSC			8	0.8919	0.7650	0.2681	0.3952
		SNV			8	0.8887	0.7575	0.2720	0.4016
		SG			8	0.7339	0.3900	0.4206	0.6369
		MC		9	0.8858	0.7374	0.2755	0.4179
	1D CAE + MLR			0.9560	**0.9359**	0.1703	**0.2093**

**Notes.**

The highest R^2^ value and the lowest RMSE value of each target were bolded to increase readability.

LV The number of Latent Variables PC The number of Principal Components

The MP5 dataset was employed in another experiment, and the results are presented in Table 6. The proposed method demonstrates superior performance, as evidenced by the higher R^2^ values for all targets in the calibration set. Similar to the M5 dataset, the proposed method outperforms conventional methods when predicting oil and starch content, as it yields higher R^2^ values and lower RMSE values. However, when assessing the prediction of moisture and protein content, it is observed that the PLSR method yields higher R^2^ values and lower RMSE values. A notable difference is observed, particularly in the oil parameter, with an increase of 20.9% in the R^2^ metric.

**Table 6:** Obtained results for the MP5 dataset.

Target	Preprocessing method	Regressor	LV	R^2^		RMSE	
			**PC**	**Calibration**	**Prediction**	**Calibration**	**Prediction**
Moisture	Original	PLSR	10	0.9243	0.8270	0.1039	0.1571
			MSC		6	0.7772	0.6167	0.1783	0.2339
			SNV		6	0.7735	0.6065	0.1798	0.2370
			SG		10	0.9241	0.8270	0.1041	0.1571
			MC	6	0.9008	**0.8276**	0.1190	**0.1569**
		Original	PCR	9	0.8493	0.6794	0.1467	0.2140
		MSC			9	0.7723	0.6005	0.1803	0.2388
		SNV			9	0.7687	0.5835	0.1817	0.2439
		SG			9	0.8490	0.6793	0.1467	0.2140
		MC		9	0.8988	0.7975	0.1201	0.1700
	1D CAE + MLR			**0.9605**	0.7851	**0.0755**	0.1725
Oil	Original	PLSR	8	0.8142	0.6027	0.0758	0.1108
			MSC		7	0.7948	0.6234	0.0796	0.1079
			SNV		7	0.7951	0.6205	0.0796	0.1083
			SG		8	0.8141	0.6028	0.0758	0.1108
			MC	10	0.8561	0.6177	0.0667	0.1087
		Original	PCR	9	0.7893	0.5810	0.0807	0.1138
		MSC			9	0.7846	0.6059	0.0816	0.1104
		SNV			9	0.7842	0.6025	0.0817	0.1109
		SG			9	0.7892	0.5808	0.0807	0.1139
		MC		9	0.7637	0.509	0.0855	0.1231
	1D CAE + MLR			**0.9245**	**0.7537**	**0.0457**	**0.1002**
Protein	Original	PLSR	10	0.9485	0.8623	0.1123	0.1838
			MSC		10	0.9483	**0.8960**	0.1126	**0.1597**
			SNV		10	0.9481	0.8939	0.1128	0.1613
			SG		10	0.9483	0.8620	0.1126	0.1840
			MC	10	0.9553	0.8787	0.1047	0.1725
		Original	PCR	8	0.8688	0.7392	0.1794	0.2530
		MSC			9	0.9034	0.7360	0.1539	0.2545
		SNV			9	0.9035	0.7318	0.1538	0.2565
		SG			8	0.8687	0.7391	0.1794	0.2530
		MC		9	0.9188	0.8376	0.1411	0.1996
	1D CAE + MLR			**0.9811**	0.8725	**0.0684**	0.1743
Starch	Original	PLSR	10	0.8936	0.7349	0.2659	0.4199
			MSC		9	0.8908	0.7813	0.2694	0.3813
			SNV		10	0.8930	0.7684	0.2667	0.3924
			SG		10	0.8933	0.7352	0.2663	0.4196
			MC	9	0.8978	0.7363	0.2606	0.4187
		Original	PCR	9	0.7363	0.3423	0.4188	0.6614
		MSC			9	0.7880	0.4174	0.3754	0.6225
		SNV			9	0.7839	0.4139	0.3790	0.6243
		SG			9	0.7361	0.3419	0.4189	0.6616
		MC		9	0.8566	0.6848	0.3087	0.4578
	1D CAE + MLR			**0.9622**	**0.7994**	**0.1578**	**0.3703**

**Notes.**

The highest R^2^ value and the lowest RMSE value of each target were bolded to increase readability.

LV The number of Latent Variables PC The number of Principal Components

The MP6 dataset was utilized in the final experiment, and the test procedure was applied in the same manner as in previous experiments. The obtained results are presented in Table 7. Although the R^2^ values were lower than those obtained in the M5 and MP5 datasets, the proposed method showed improved performance on all targets in the MP6 dataset, as evidenced by the higher R^2^ values and lower RMSE values. Additionally, when analyzing the prediction of the oil and starch parameters, it was found that conventional methods were unable to establish a viable model, as the R^2^ value was below 0.7.

**Table 7:** Obtained results for the MP6 dataset.

Target	Preprocessing method	Regressor	LV	R^2^		RMSE	
			**PC**	**Calibration**	**Prediction**	**Calibration**	**Prediction**
Moisture	Original	PLSR	8	0.8880	0.7568	0.1264	0.1863
			MSC		5	0.7089	0.5165	0.2039	0.2628
			SNV		5	0.7007	0.5081	0.2067	0.2650
			SG		8	0.8879	0.7569	0.1265	0.1863
			MC	7	0.8771	0.7705	0.1324	0.1810
		Original	PCR	8	0.7831	0.6226	0.1760	0.2321
		MSC			9	0.7400	0.5225	0.1927	0.2611
		SNV			9	0.7326	0.4963	0.1950	0.2682
		SG			8	0.7831	0.6224	0.1760	0.2322
		MC		7	0.8560	0.7500	0.1434	0.1889
	1D CAE + MLR			**0.9530**	**0.8254**	**0.0824**	**0.1555**
Oil	Original	PLSR	9	0.8274	0.6236	0.0730	0.1079
			MSC		10	0.8448	0.6647	0.0693	0.1018
			SNV		10	0.8441	0.6487	0.0694	0.1042
			SG		9	0.8272	0.6234	0.0731	0.1079
			MC	10	0.8656	0.6819	0.0644	0.0992
		Original	PCR	9	0.7910	0.5541	0.0804	0.1174
		MSC			7	0.7597	0.5984	0.0862	0.1114
		SNV			7	0.7571	0.5900	0.0866	0.1126
		SG			9	0.7910	0.5539	0.0804	0.1175
		MC		8	0.7259	0.5108	0.0920	0.1230
	1D CAE + MLR			**0.9096**	**0.8199**	**0.0500**	**0.0857**
Protein	Original	PLSR	10	0.9502	0.8863	0.1104	0.1670
			MSC		10	0.9446	0.8950	0.1165	0.1605
			SNV		10	0.9442	0.8934	0.1169	0.1617
			SG		10	0.9502	0.8858	0.1105	0.1673
			MC	10	0.9532	0.8820	0.1071	0.1701
		Original	PCR	9	0.8737	0.7658	0.1760	0.2397
		MSC			9	0.9095	0.7765	0.1490	0.2342
		SNV			9	0.9099	0.7784	0.1487	0.2331
		SG			9	0.8737	0.7662	0.1760	0.2395
		MC		9	0.9088	0.7884	0.1495	0.2279
	1D CAE + MLR			**0.9681**	**0.8995**	**0.0889**	**0.1548**
Starch	Original	PLSR	10	0.8957	0.6287	0.2633	0.4969
			MSC		8	0.8731	0.6616	0.2904	0.4744
			SNV		8	0.8679	0.6644	0.2964	0.4724
			SG		10	0.8956	0.6272	0.2635	0.4979
			MC	9	0.8815	0.6697	0.2807	0.4686
		Original	PCR	9	0.7775	0.4936	0.3846	0.5803
		MSC			9	0.8312	0.5568	0.3350	0.5429
		SNV			9	0.8258	0.5528	0.3403	0.5453
		SG			9	0.7777	0.4944	0.3844	0.5798
		MC		9	0.8284	0.4976	0.3378	0.5780
	1D CAE + MLR			**0.9002**	**0.7988**	**0.2563**	**0.3709**

**Notes.**

The highest R^2^ value and the lowest RMSE value of each target were bolded to increase readability.

LV The number of Latent Variables PC The number of Principal Components

## Discussion

As mentioned before, preprocessing is an inevitable stage of NIRS modeling techniques. According to Tables 5, 6 and 7, four different preprocessing methods have yielded higher scores on different metrics, confirming this hypothesis. However, the inherent trial and error have led researchers to look for new preprocessing methods. Although some innovative methods have been proposed, they have not been widely used (Helin et al., 2022; Xu et al., 2022). But still, DL-based approaches give promising results. 1D-CAE and MLR combination offers a new approach to this problem.

**Figure 7:**
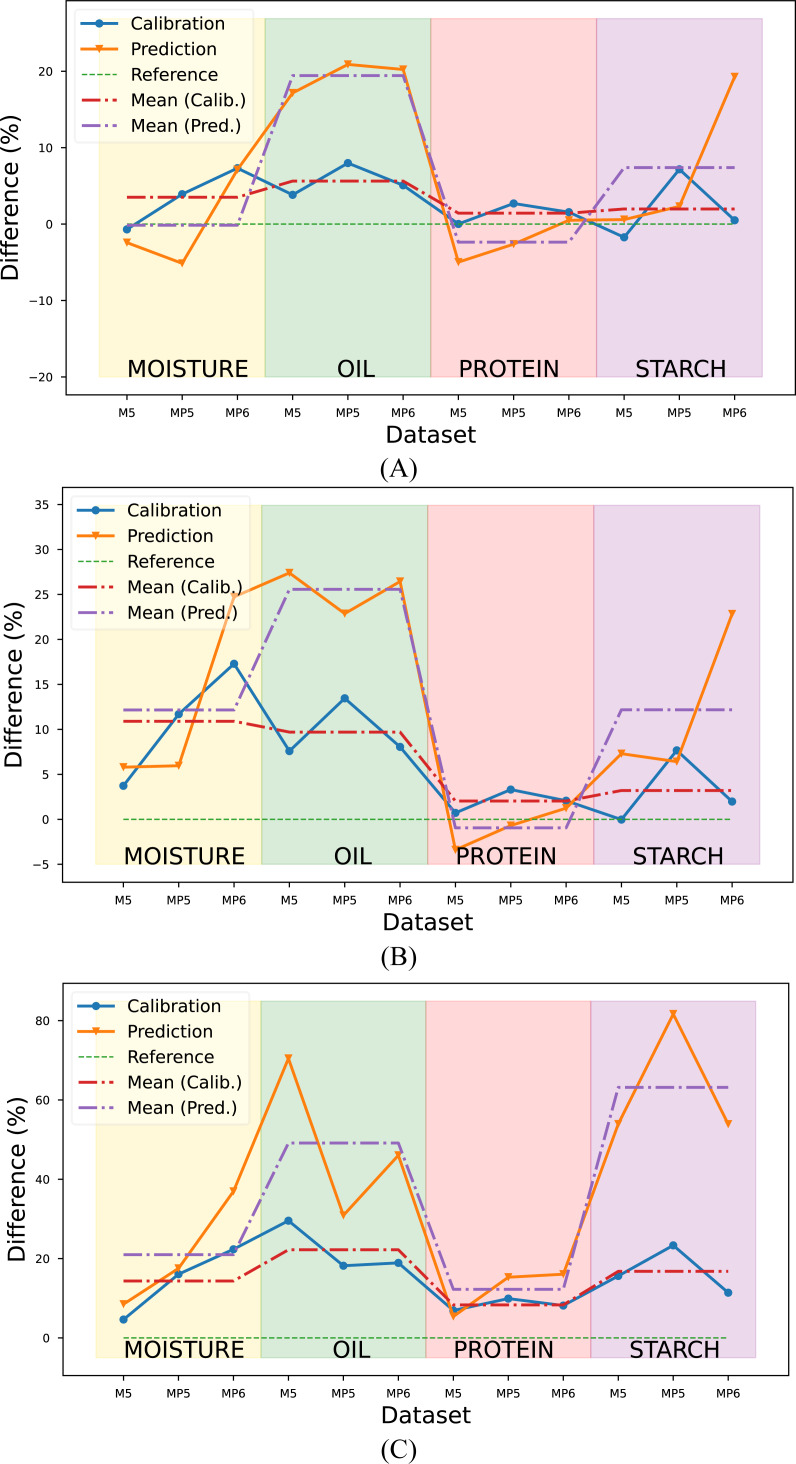
Comparison of the proposed method with conventional methods according to the R^2^ metric. Reference value (green line) corresponds (A) Conventional method combination with the highest R^2^ value, (B) the mean R^2^ value of PLSR combinations, (C) the mean R^2^ value of PCR combinations.

The R^2^ values obtained with the proposed method in each target parameter and dataset were calculated as a percentage and illustrated in Fig. 7. The RMSE metric was not considered in the evaluation since it provided results consistent with the R^2^ metric.

Upon a comprehensive evaluation of the results in Tables 5, 6 and 7, it was observed that the proposed method yielded a 3.52% increase in the mean R^2^ metric in the calibration set, compared to the highest R^2^ value obtained with conventional method combinations in the moisture parameter. However, a slightly lower value of 0.14% was obtained in the prediction set. The R^2^ values were calculated for the combinations generated using the PLSR method, and the mean R^2^ value was determined and compared with the mean R^2^ value obtained with the proposed method. Results indicated that the proposed method yielded a 10.9% and 12.16% improvement in R^2^ values for the calibration and prediction sets, respectively, compared to the PLSR method combinations. The comparison was also made with the PCR method for the prediction of moisture content. Results indicated that the proposed method yielded an improvement of 14.34% and 20.99% in R^2^ values for the calibration and prediction sets, respectively when compared to PCR combinations.

The evaluation was also performed for the oil parameter by utilizing the results from three datasets. The proposed method was compared to the conventional method combinations with the highest R^2^ value, the mean R^2^ value of PLSR combinations, and the mean R^2^ value of PCR combinations. The results showed that the proposed method yielded an improvement of 5.63% and 19.43% in R^2^ values for the calibration and prediction sets, respectively when compared to the conventional method with the highest R^2^ value. Additionally, the proposed method showed a 9.70% and 25.57% improvement in R^2^ values for the calibration and prediction sets, respectively, when compared to the mean R^2^ values of the PLSR combinations and 22.22% and 49.13% improvement in R^2^ values for the calibration and prediction sets respectively when compared to the mean R^2^ values of the PCR combinations.

In predicting the third target, protein content, the proposed method yielded a 1.43% improvement in the calibration set compared to the conventional method combination with the highest R^2^ value. Conversely, a 2.37% decline was noted in the prediction set. Similarly, compared to the mean R^2^ value of the PLSR combinations, the proposed method demonstrated a 2.03% enhancement in the calibration set and a 0.95% decline in the prediction set. The proposed method revealed an 8.33% and 12.25% increase compared to the mean R^2^ value of the PCR combinations in the calibration and prediction set, respectively.

The proposed method for determining the starch content of corn samples was found to be highly efficacious, as evidenced by its significant improvement in performance when compared to PLSR and PCR combinations. Specifically, the proposed method exhibited an improvement of 1.98%, 7.40%, 3.20%, 12.18%, 16.78%, and 63.16% to the conventional method combination with the highest R^2^ value, the mean R^2^ value of PLSR combinations, and the mean R^2^ value of PCR combinations, respectively.

**Figure 8:**
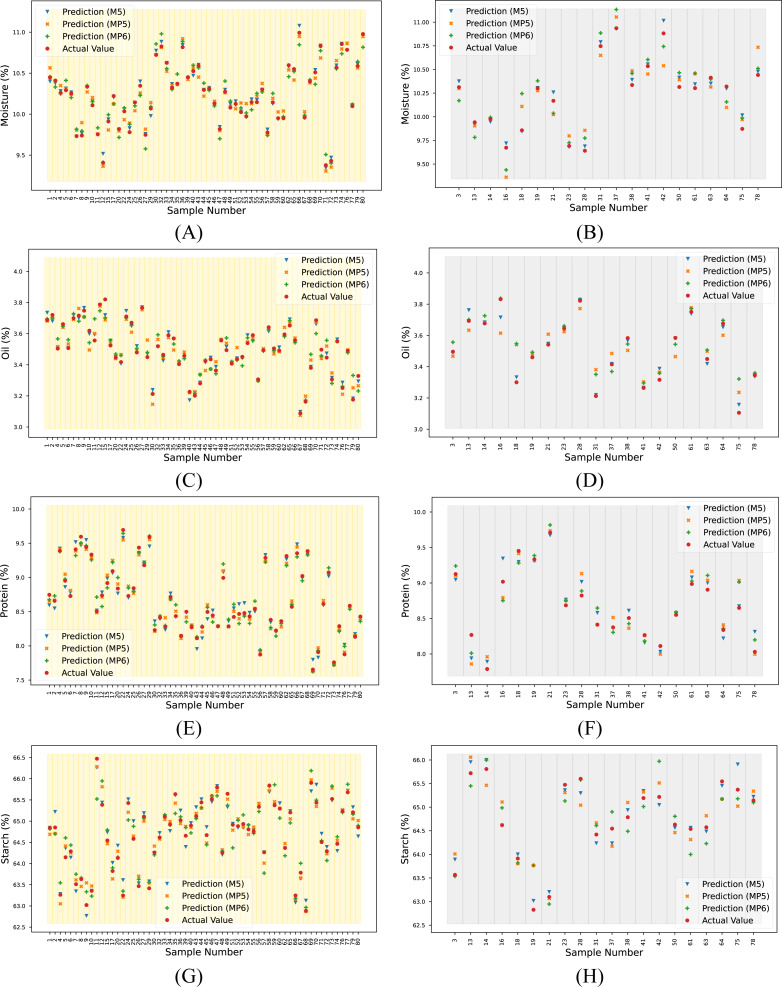
Actual and predicted outputs using the proposed method for each target. (A) Moisture (calibration), (B) moisture (prediction), (C) oil (calibration), (D) oil (prediction), (E) protein (calibration), (F) protein (prediction), (G) starch (calibration) and (H) starch (prediction).

For the overall assessment, the proposed method yielded higher R^2^ values, especially when predicting the oil and starch parameters for each dataset. The reference and predicted output for each target and spectrum are given in Table S2. These data are visualized in Fig. 8.

Table 8 presents a compilation and comparison of studies in the literature that utilize the corn dataset with the proposed method. Bian et al. (2016) employed four different PLSR-based methods for estimating protein parameters utilizing the MP6 dataset. These methods were found to enhance the performance of the traditional PLSR method. Upon comparing the four methods utilized in the study, it was observed that the proposed 1D-CAE+MLR method demonstrated superior results with higher R^2^ and lower RMSE values. Yuanyuan & Zhibin (2018) have proposed four different models based on neural networks and deep learning to estimate four parameters of the corn dataset. The dataset used in this study was not specified. Upon examination of the graph provided in the study, it is inferred that the MP5 dataset was used, and the comparison was made accordingly. It was observed that the proposed method gave a higher R^2^ value in the protein and starch parameters compared to the methods used in this study, while it gave a lower R^2^ value in the moisture and oil parameters. Fatemi, Singh & Kamruzzaman (2022) developed wavelength selection-based models to predict four parameters of corn seeds using the M5 dataset. They identified a specific wavelength range for each parameter. When we compare our result with this study, our method gives a higher R^2^ value for the oil parameter, but wavelength selection-based models give a higher R^2^ value for the other three parameters. According to these studies, it can be seen that competitive results are obtained with the proposed method.

To confirm the statistical validity of the results obtained with the proposed method, a *t*-test was performed. Based on the results of the *t*-test, it was determined that all of the results obtained with the proposed method fell within the 99.9% confidence interval (*p*-value < 0.001).

Another highlight of this study is that although there are no significant changes in the obtained spectra due to the measurement of the same sample with different instruments, the success of conventional chemometric methods decreases significantly. This shows that the success of chemometric methods is spectrum dependent, as is the case with preprocessing methods.

Deep learning models require a larger quantity of samples for training compared to traditional neural networks to construct an accurate model. Failure to do so results in underfitting, where the model is unable to capture the underlying pattern of the data. As the generation of NIR datasets and their corresponding reference values is a laborious process, such datasets often have a limited number of samples, as in the corn dataset. This situation represents the limitations of the proposed method as well as other DL models.

**Table 8:** The comparison of the studies using the corn dataset.

**Reference**	**Method**	**M5 dataset**				**MP5 dataset**				**MP6 dataset**			
		**Moisture**	**Oil**	**Protein**	**Starch**	**Moisture**	**Oil**	**Protein**	**Starch**	**Moisture**	**Oil**	**Protein**	**Starch**
Bian et al. (2016) ^a^	PLS	–	–	–	–	–	–	–	–	–	–	0.8815	–
		MCUEV-PLS	–	–	–	–	–	–	–	–	–	–	0.8870	–
		RT-PLS	–	–	–	–	–	–	–	–	–	–	0.8913	–
	VS-PLS	–	–	–	–	–	–	–	–	–	–	0.8932	–
Yuanyuan & Zhibin (2018) ^b^	ECNN	–	–	–	–	0.9471	0.8079	0.8172	0.7278	–	–	–	–
		CNN	–	–	–	–	0.9339	0.7545	0.8082	0.6988	–	–	–	–
		BP-ANN	–	–	–	–	0.8813	0.6109	0.7848	0.6552	–	–	–	–
	PLS	–	–	–	–	0.9143	0.7052	0.7391	0.6932	–	–	–	–
Fatemi, Singh & Kamruzzaman (2022)	WS-MLR	0.9999	0.88	0.99	0.96	–	–	–	–	–	–	–	–
Proposed method		0.9716	0.9631	0.9012	0.9359	0.7851	0.7537	0.8725	0.7994	0.8254	0.8199	0.8995	0.7988

**Notes.**

a The results obtained in this study were reported according to the R (correlation coefficient) metric, and these values have been converted to the R^2^ metric to ensure compliance.

b In the study, the used dataset was not specified, and this table was prepared considering that the used dataset was MP5, according to the graph given in the study.

Abbreviations MCUEV-PLS Monte Carlo uninformative variable elimination-partial linear regression RT-PLS Randomization test-partial linear regression VS-BPLS Variable space boosting-partial linear regression ECNN Ensemble convolutional neural networks CNN Convolutional neural networks BP-ANN Backpropagation—Artificial Neural Networks WS-MLR Wavelength Selection—Multiple Linear Regression

## Conclusions

A one-dimensional convolutional autoencoder-based NIR modeling technique is proposed to assess the quality parameters of corn kernels. With 1D-CAE, the need for preprocessing the spectrum, which is the common point of chemometric methods, is eliminated. The proposed method was tested on three different spectra obtained from different devices in the corn dataset, showing that our results are device independent. The results indicate that our method has superior performance over common preprocessing and chemometric method combinations according to R^2^ and RMSE metrics, especially in oil and starch parameters. Our method provides a reliable model that ensures fast and precise analysis in near-infrared spectroscopy. Future investigations should focus on applying the proposed method to calibration transfer.

##  Supplemental Information

10.7717/peerj-cs.1266/supp-1 Supplemental Information 1 Supplemental Figures and Table

## Additional Information and Declarations

### Data Availability

The following information was supplied regarding data availability: The raw data and code are available at GitHub: https://github.com/ozcan412/1D-CAE_MLR.
