# Supplementary material for: Detection of protein, starch, oil, and moisture content of corn kernels using one-dimensional convolutional autoencoder and near-infrared spectroscopy
Source: PeerJ Comput Sci. 2023 Mar 9;9:e1266. doi: 10.7717/peerj-cs.1266 (PMC10280583; doi:10.7717/peerj-cs.1266)
Supplement: Supplemental Information 1 [file peerj-cs-09-1266-s001.docx]

| Input spectrum | 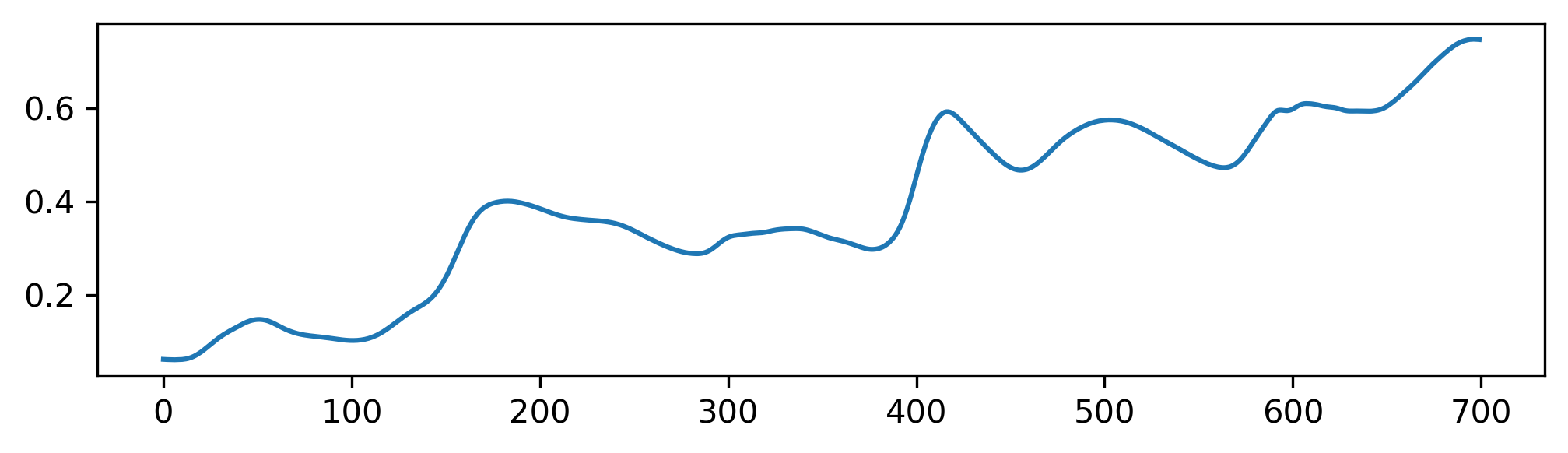 |
| --- | --- |
| First convolutional layer | 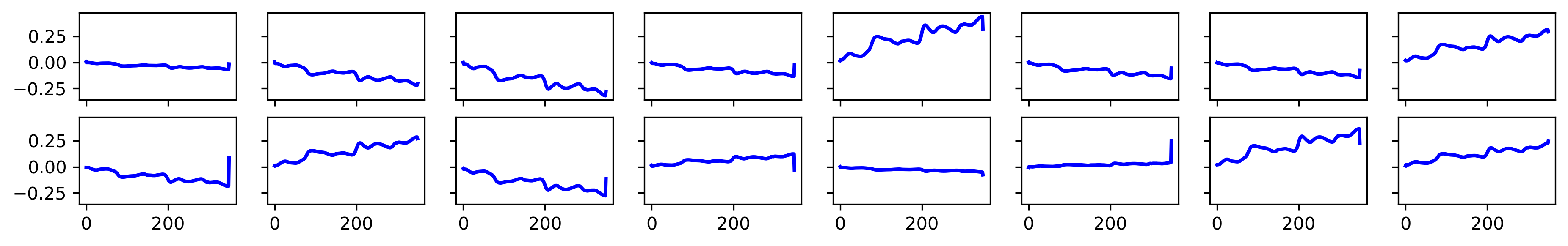 |
| Second convolutional layer | 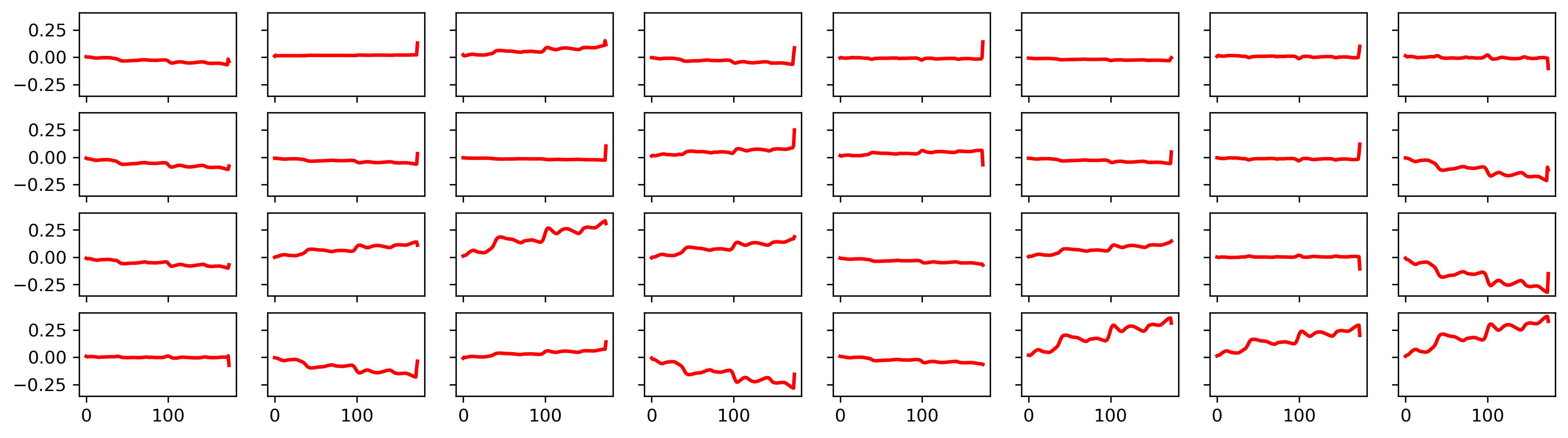 |

**Figure S1.** Visualization of a sample spectrum from the calibration set and its feature maps from the M5 dataset

| Input spectrum | 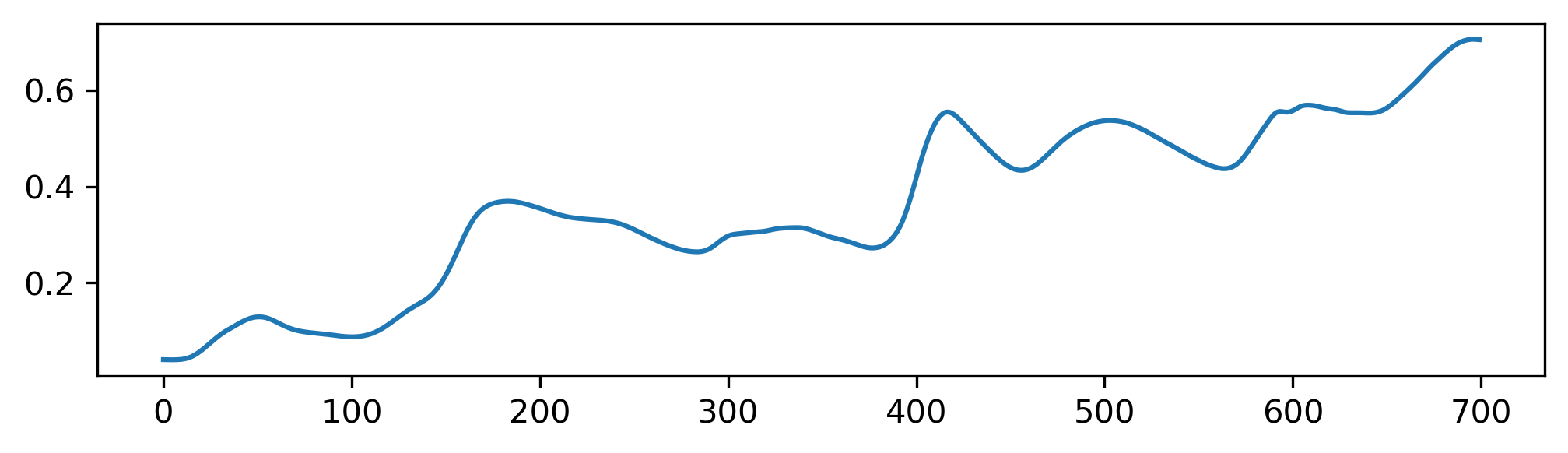 |
| --- | --- |
| First convolutional layer | 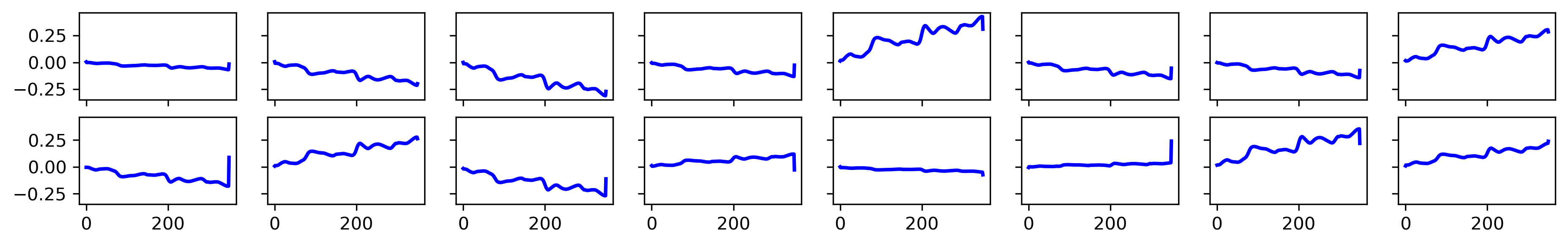 |
| Second convolutional layer | 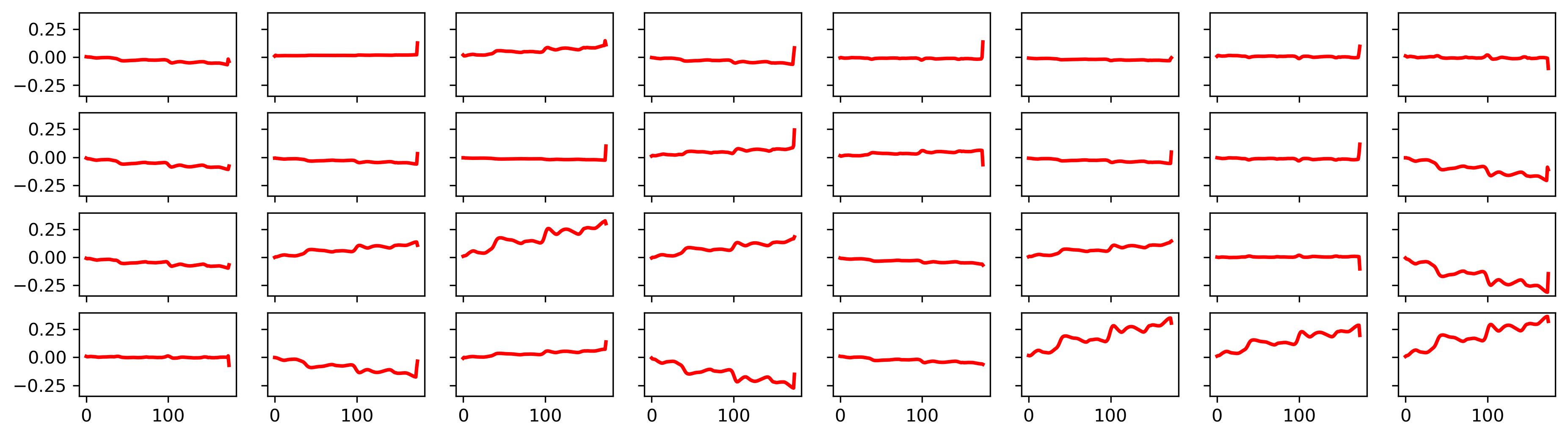 |

**Figure S2.** Visualization of a sample spectrum from the prediction set and its feature maps from the M5 dataset

| Input spectrum | 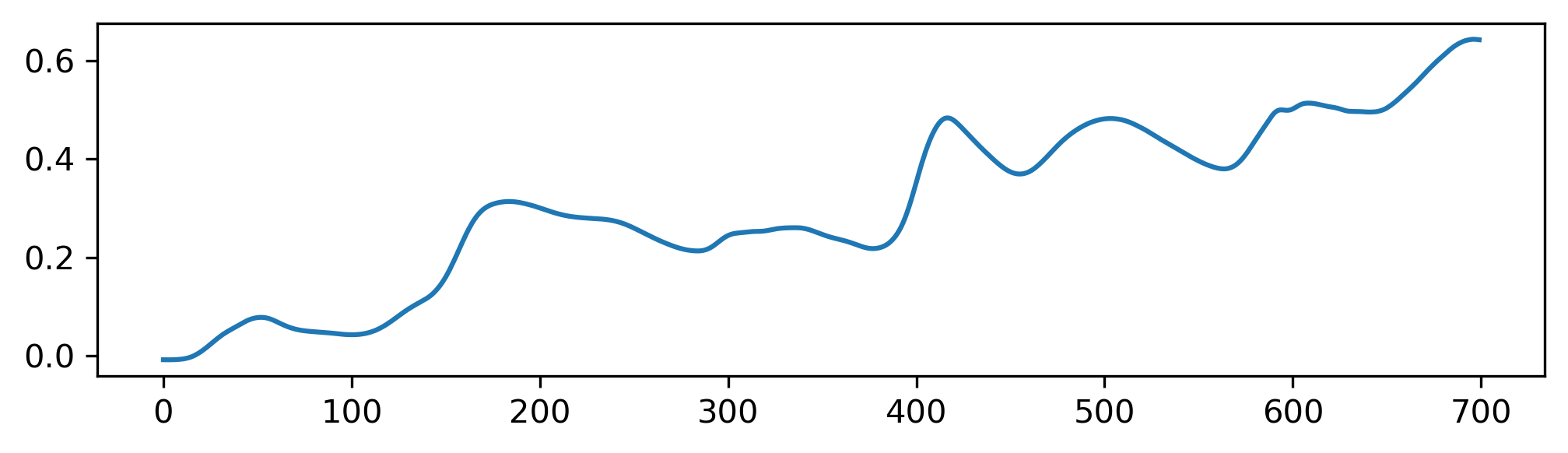 |
| --- | --- |
| First convolutional layer | 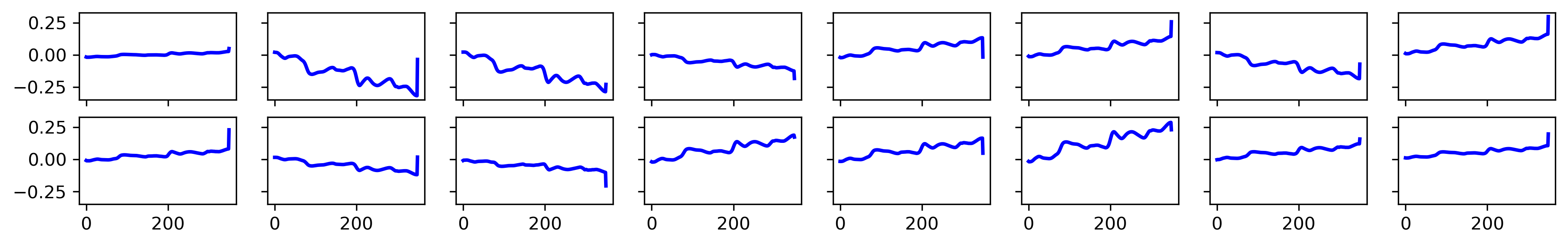 |
| Second convolutional layer | 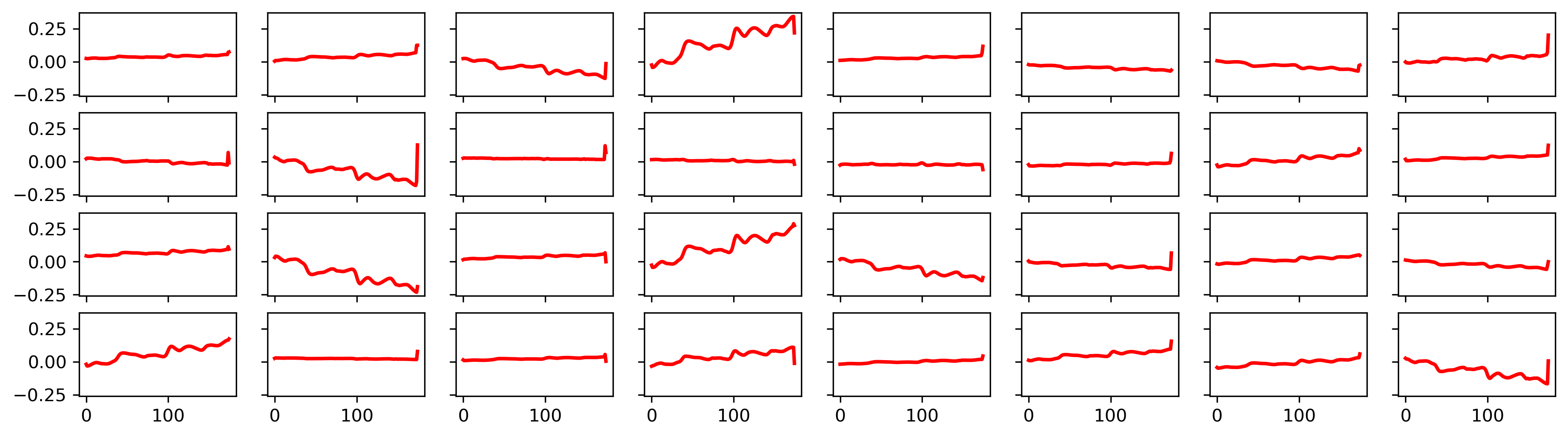 |

**Figure S3.** Visualization of a sample spectrum from the calibration set and its feature maps from the MP5 dataset

| Input spectrum | 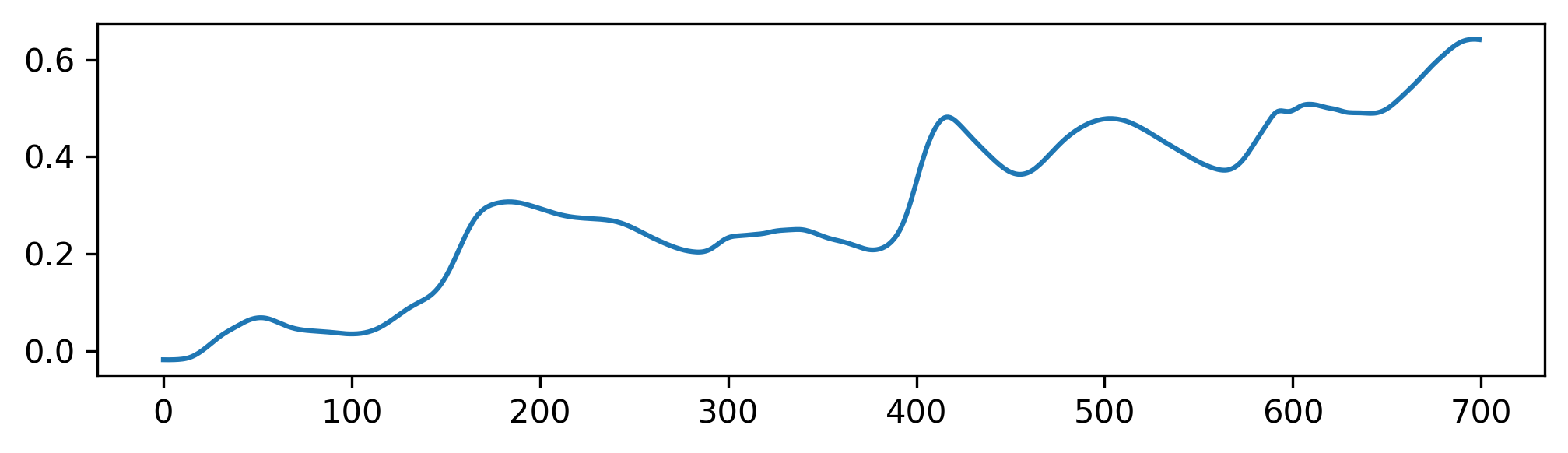 |
| --- | --- |
| First convolutional layer | 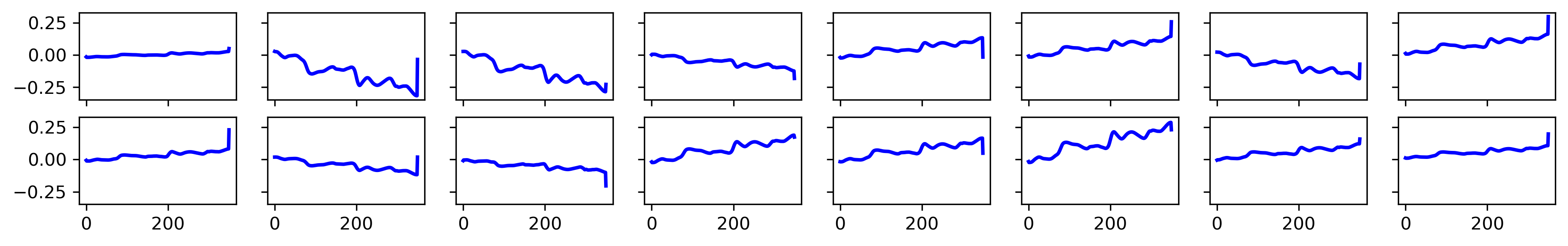 |
| Second convolutional layer | 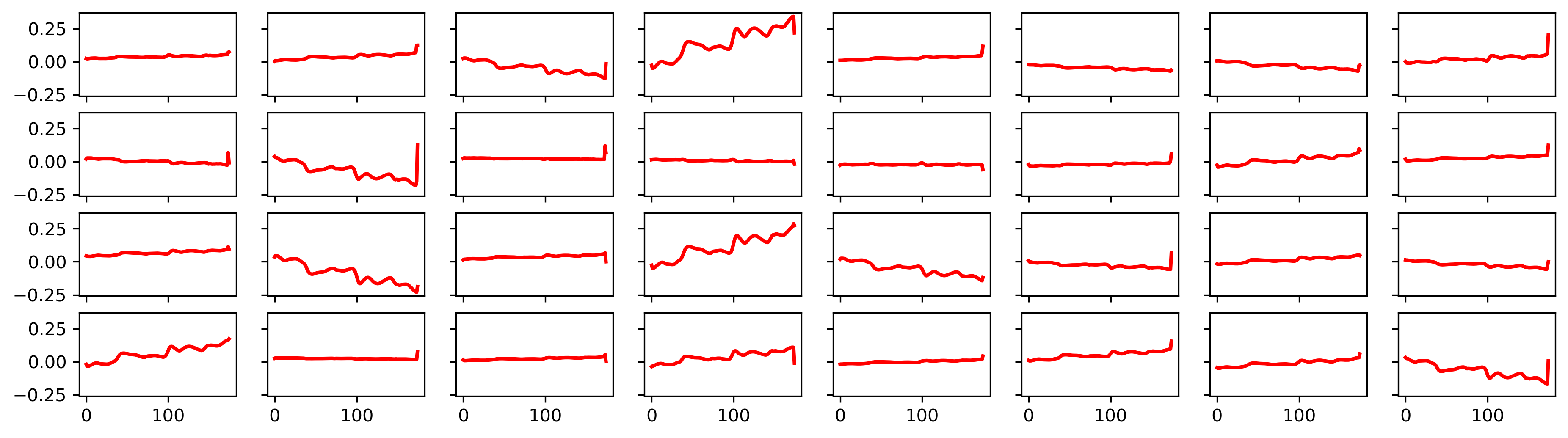 |

**Figure S4.** Visualization of a sample spectrum from the prediction set and its feature maps from the MP5 dataset

| Input spectrum | 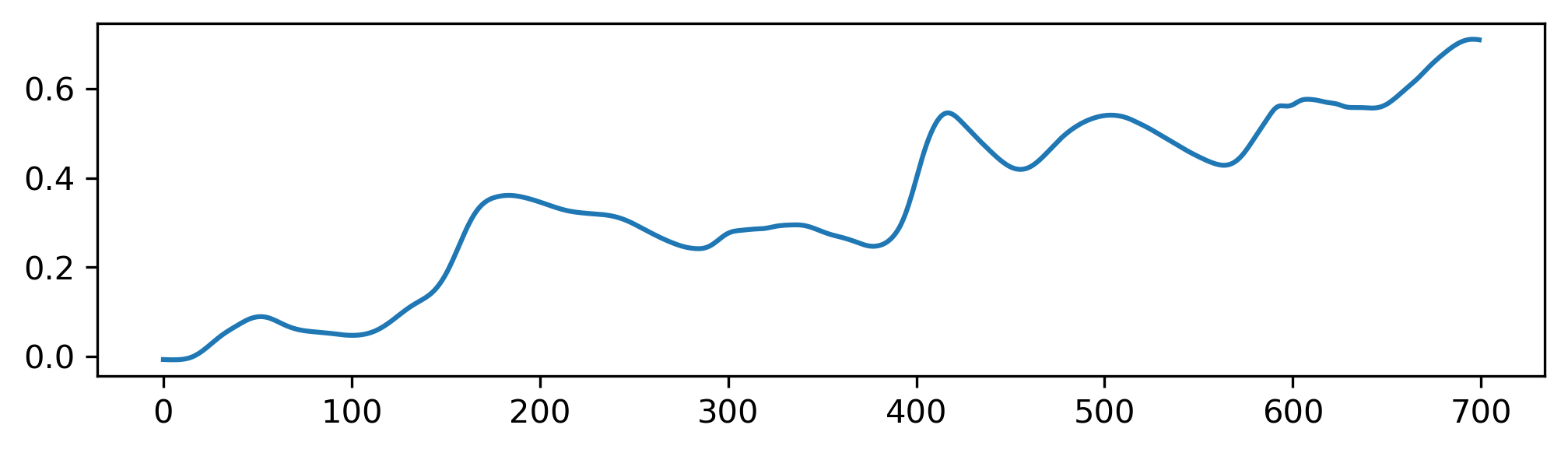 |
| --- | --- |
| First convolutional layer | 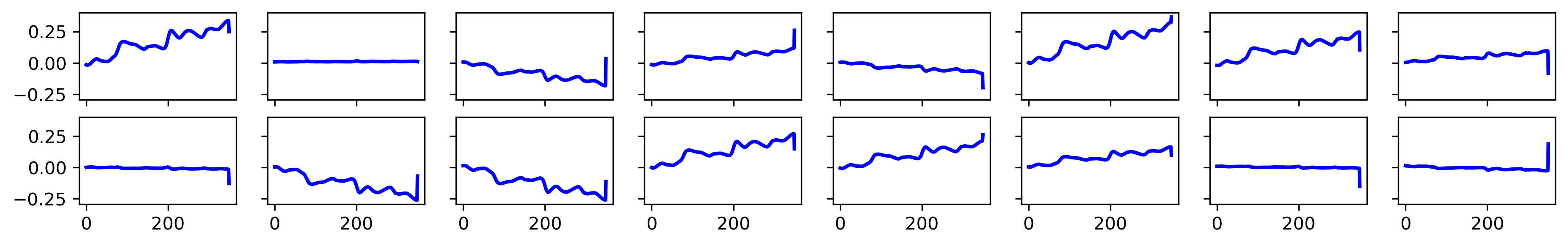 |
| Second convolutional layer | 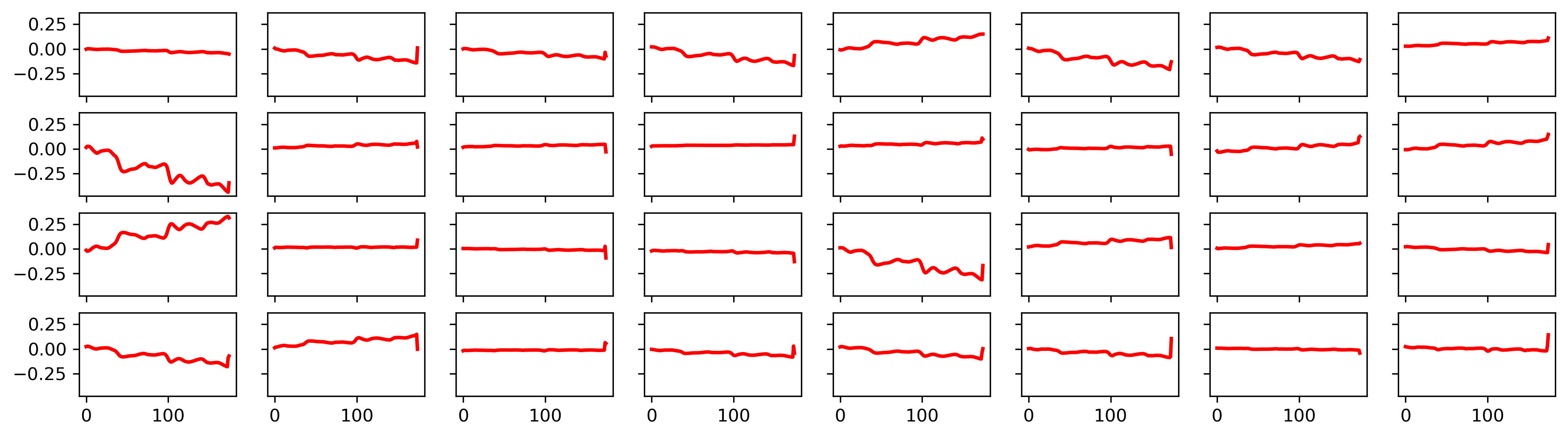 |

**Figure S5.** Visualization of a sample spectrum from the calibration set and its feature maps from the MP6 dataset

| Input spectrum | 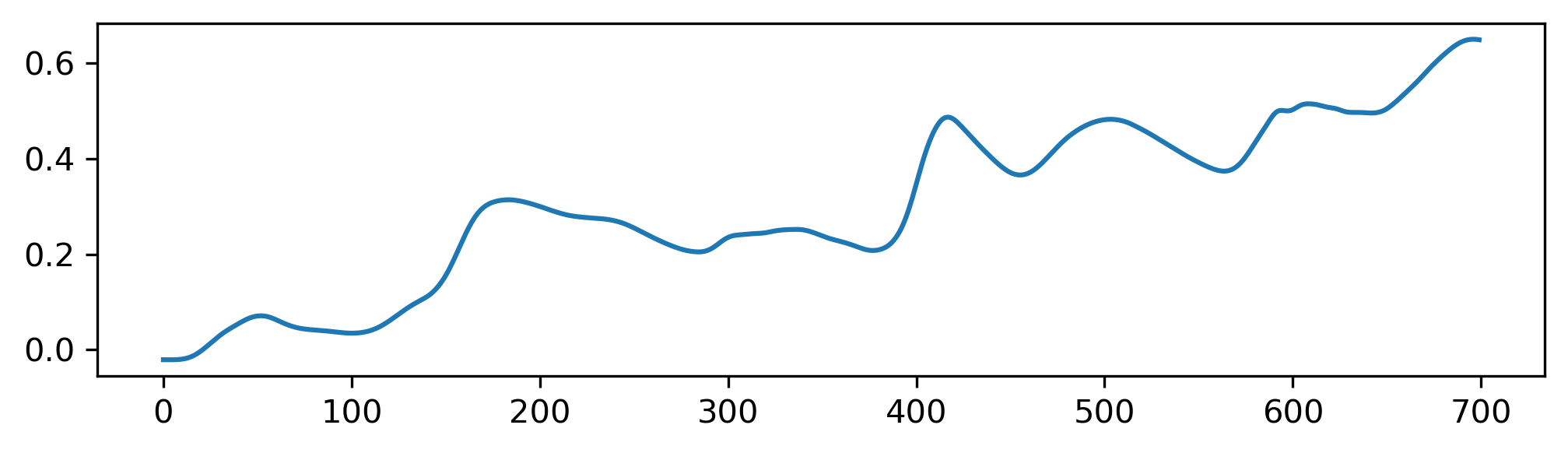 |
| --- | --- |
| First convolutional layer | 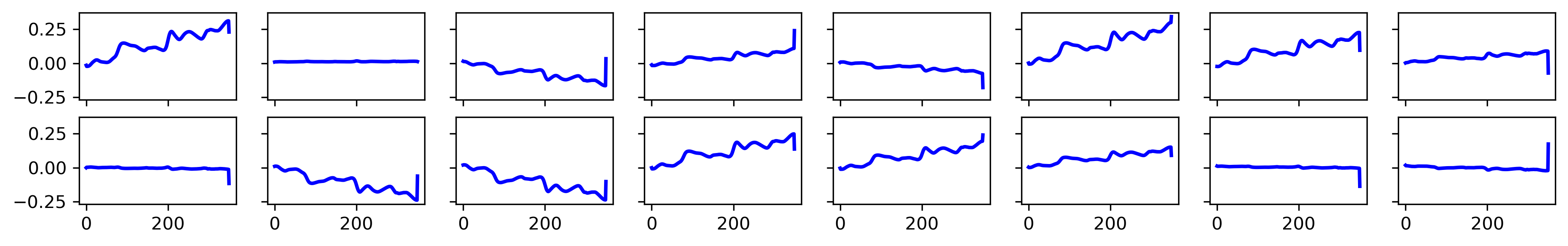 |
| Second convolutional layer | 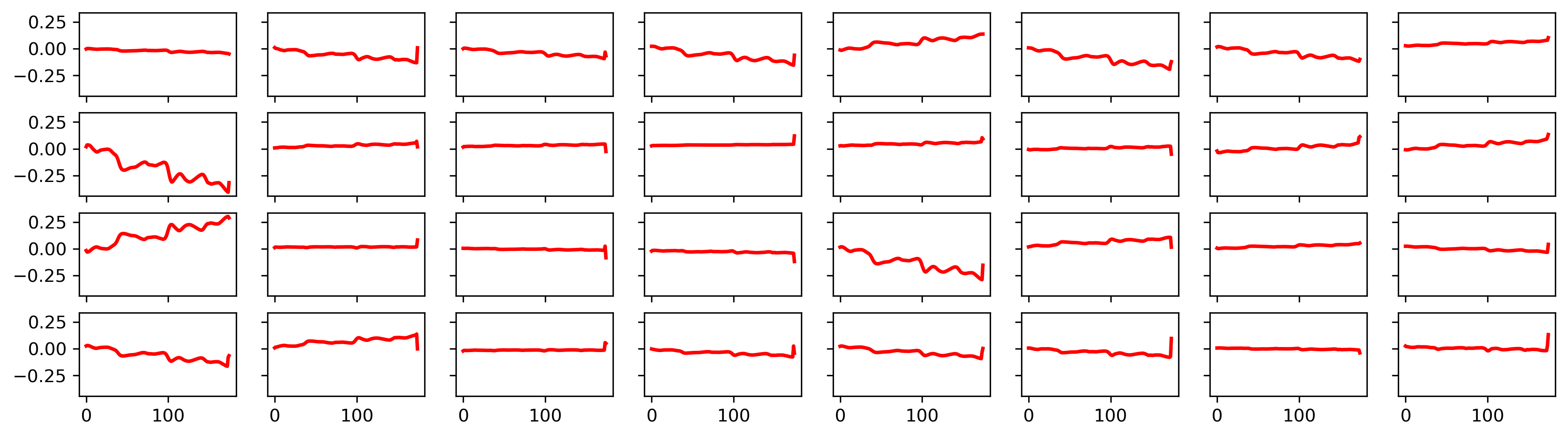 |

**Figure S6.** Visualization of a sample spectrum from the prediction set and its feature maps from the MP6 dataset

**Table S1.** Lookup table used for hyperparameter tuning

| **Hyperparameter** | **Search space** | **Selected value** |
| --- | --- | --- |
| Kernel size | 1,3,5,7,9 | 5 for encoder, 3 for decoder |
| Batch size | 4,8,10,16 | 10 |
| Latent variables | 2,4,8,16,32 | 32 |

**Table S2a.** Obtained results with the proposed method for moisture parameter.

| **No** | **Ref.** | **Pred.**  **(M5)** | **Pred.**  **(MP5)** | **Pred.**  **(MP6)** | **Set** | **No** | **Ref.** | **Pred.**  **(M5)** | **Pred.**  **(MP5)** | **Pred.**  **(MP6)** | **Set** |
| --- | --- | --- | --- | --- | --- | --- | --- | --- | --- | --- | --- |
| **1** | 10.448 | 10.3984 | 10.5649 | 10.4561 | Cal. | **41** | 10.535 | 10.5547 | 10.4519 | 10.6045 | Pred. |
| **2** | 10.409 | 10.375 | 10.4078 | 10.3311 | Cal. | **42** | 10.882 | 11.0156 | 10.5397 | 10.7432 | Pred. |
| **3** | 10.313 | 10.375 | 10.2989 | 10.1709 | Pred. | **43** | 10.592 | 10.6016 | 10.4514 | 10.5654 | Cal. |
| **4** | 10.26 | 10.2812 | 10.3486 | 10.249 | Cal. | **44** | 10.296 | 10.2969 | 10.2202 | 10.3779 | Cal. |
| **5** | 10.292 | 10.3125 | 10.3068 | 10.4111 | Cal. | **45** | 10.311 | 10.3203 | 10.316 | 10.29 | Cal. |
| **6** | 10.253 | 10.2656 | 10.244 | 10.2021 | Cal. | **46** | 10.13 | 10.1172 | 10.1569 | 10.1006 | Cal. |
| **7** | 9.732 | 9.72656 | 9.80605 | 9.81543 | Cal. | **47** | 9.817 | 9.84375 | 9.79918 | 9.7002 | Cal. |
| **8** | 9.739 | 9.76562 | 9.89595 | 9.79395 | Cal. | **48** | 10.273 | 10.2969 | 10.2605 | 10.4033 | Cal. |
| **9** | 10.335 | 10.3281 | 10.2706 | 10.3486 | Cal. | **49** | 10.08 | 10.125 | 10.0961 | 10.1592 | Cal. |
| **10** | 10.108 | 10.1562 | 10.2022 | 10.1514 | Cal. | **50** | 10.315 | 10.4141 | 10.3908 | 10.4658 | Pred. |
| **11** | 9.754 | 9.75 | 9.75783 | 9.83301 | Cal. | **51** | 10.126 | 10.1562 | 10.0658 | 10.1162 | Cal. |
| **12** | 9.407 | 9.51562 | 9.36336 | 9.41113 | Cal. | **52** | 10.025 | 10.0312 | 10.1363 | 10.0752 | Cal. |
| **13** | 9.942 | 9.91406 | 9.90379 | 9.78223 | Pred. | **53** | 9.973 | 9.96094 | 10.1285 | 10.0146 | Cal. |
| **14** | 9.978 | 9.94531 | 9.97765 | 9.99316 | Pred. | **54** | 10.146 | 10.1797 | 10.1279 | 10.0537 | Cal. |
| **15** | 9.911 | 9.9375 | 9.80678 | 9.99316 | Cal. | **55** | 10.146 | 10.1719 | 10.2156 | 10.2529 | Cal. |
| **16** | 9.673 | 9.71875 | 9.36113 | 9.43848 | Pred. | **56** | 10.301 | 10.2969 | 10.3746 | 10.2705 | Cal. |
| **17** | 10.221 | 10.2031 | 10.1239 | 10.126 | Cal. | **57** | 9.776 | 9.8125 | 9.74947 | 9.74121 | Cal. |
| **18** | 9.857 | 9.84375 | 10.1096 | 10.2451 | Pred. | **58** | 10.143 | 10.125 | 10.193 | 10.2529 | Cal. |
| **19** | 10.302 | 10.3047 | 10.276 | 10.3799 | Pred. | **59** | 9.95 | 9.9375 | 10.0266 | 9.9502 | Cal. |
| **20** | 9.818 | 9.8125 | 9.79402 | 9.71582 | Cal. | **60** | 9.95 | 9.94531 | 10.0387 | 9.96973 | Cal. |
| **21** | 10.169 | 10.2578 | 10.0248 | 10.0361 | Pred. | **61** | 10.303 | 10.3438 | 10.454 | 10.46 | Pred. |
| **22** | 10.034 | 10.0625 | 9.93208 | 10.0752 | Cal. | **62** | 10.597 | 10.5781 | 10.5392 | 10.458 | Cal. |
| **23** | 9.691 | 9.67969 | 9.79808 | 9.72559 | Pred. | **63** | 10.413 | 10.3516 | 10.315 | 10.3994 | Pred. |
| **24** | 9.78 | 9.82812 | 9.89207 | 9.87598 | Cal. | **64** | 10.32 | 10.2969 | 10.0983 | 10.1572 | Pred. |
| **25** | 10.143 | 10.1406 | 10.0413 | 10.1006 | Cal. | **65** | 10.55 | 10.5156 | 10.4159 | 10.5205 | Cal. |
| **26** | 10.346 | 10.3984 | 10.2579 | 10.2275 | Cal. | **66** | 10.993 | 11.0781 | 10.9472 | 10.8467 | Cal. |
| **27** | 9.748 | 9.75781 | 9.81731 | 9.57715 | Cal. | **67** | 9.958 | 9.95312 | 10.0293 | 9.98535 | Cal. |
| **28** | 9.641 | 9.6875 | 9.8568 | 9.77441 | Pred. | **68** | 10.398 | 10.3984 | 10.3809 | 10.4189 | Cal. |
| **29** | 10.069 | 9.97656 | 10.1405 | 10.0908 | Cal. | **69** | 10.509 | 10.5391 | 10.439 | 10.3643 | Cal. |
| **30** | 10.723 | 10.7734 | 10.7268 | 10.8564 | Cal. | **70** | 10.831 | 10.8359 | 10.6853 | 10.7744 | Cal. |
| **31** | 10.747 | 10.7891 | 10.6492 | 10.8857 | Pred. | **71** | 9.377 | 9.34375 | 9.30562 | 9.50684 | Cal. |
| **32** | 10.826 | 10.8828 | 10.8147 | 10.9795 | Cal. | **72** | 9.43 | 9.46875 | 9.35414 | 9.40332 | Cal. |
| **33** | 10.626 | 10.625 | 10.5231 | 10.5557 | Cal. | **73** | 10.568 | 10.5938 | 10.6539 | 10.5537 | Cal. |
| **34** | 10.323 | 10.3672 | 10.3499 | 10.3018 | Cal. | **74** | 10.855 | 10.8594 | 10.795 | 10.7373 | Cal. |
| **35** | 10.371 | 10.3594 | 10.3628 | 10.4893 | Cal. | **75** | 9.872 | 10.0156 | 9.97054 | 9.9873 | Pred. |
| **36** | 10.817 | 10.8438 | 10.9202 | 10.8857 | Cal. | **76** | 10.785 | 10.8594 | 10.8652 | 10.7842 | Cal. |
| **37** | 10.936 | 10.9297 | 11.0549 | 11.1338 | Pred. | **77** | 10.097 | 10.1094 | 10.0994 | 10.1182 | Cal. |
| **38** | 10.336 | 10.3906 | 10.4864 | 10.458 | Pred. | **78** | 10.442 | 10.4766 | 10.7352 | 10.5107 | Pred. |
| **39** | 10.451 | 10.4375 | 10.4223 | 10.4482 | Cal. | **79** | 10.587 | 10.6094 | 10.5631 | 10.6416 | Cal. |
| **40** | 10.525 | 10.4688 | 10.5611 | 10.5967 | Cal. | **80** | 10.977 | 10.9609 | 10.9473 | 10.8154 | Cal. |

**Table S2b.** Obtained results with the proposed method for oil parameter.

| **No** | **Ref.** | **Pred.**  **(M5)** | **Pred.**  **(MP5)** | **Pred.**  **(MP6)** | **Set** | **No** | **Ref.** | **Pred.**  **(M5)** | **Pred.**  **(MP5)** | **Pred.**  **(MP6)** | **Set** |
| --- | --- | --- | --- | --- | --- | --- | --- | --- | --- | --- | --- |
| **1** | 3.687 | 3.73438 | 3.68088 | 3.69531 | Cal. | **41** | 3.264 | 3.26562 | 3.30252 | 3.2959 | Pred. |
| **2** | 3.72 | 3.67969 | 3.70084 | 3.70117 | Cal. | **42** | 3.316 | 3.38672 | 3.36331 | 3.3584 | Pred. |
| **3** | 3.496 | 3.48828 | 3.46677 | 3.55664 | Pred. | **43** | 3.201 | 3.21875 | 3.22726 | 3.21484 | Cal. |
| **4** | 3.504 | 3.50781 | 3.51688 | 3.56641 | Cal. | **44** | 3.28 | 3.28516 | 3.33859 | 3.33594 | Cal. |
| **5** | 3.661 | 3.65234 | 3.64072 | 3.64648 | Cal. | **45** | 3.424 | 3.41406 | 3.36301 | 3.43066 | Cal. |
| **6** | 3.507 | 3.50781 | 3.53336 | 3.56055 | Cal. | **46** | 3.435 | 3.44531 | 3.37186 | 3.37305 | Cal. |
| **7** | 3.699 | 3.71875 | 3.69064 | 3.72852 | Cal. | **47** | 3.363 | 3.38672 | 3.4194 | 3.34277 | Cal. |
| **8** | 3.716 | 3.69922 | 3.76175 | 3.67969 | Cal. | **48** | 3.556 | 3.55859 | 3.56375 | 3.56055 | Cal. |
| **9** | 3.748 | 3.76562 | 3.7081 | 3.70703 | Cal. | **49** | 3.494 | 3.51562 | 3.53842 | 3.57227 | Cal. |
| **10** | 3.619 | 3.59766 | 3.49484 | 3.54199 | Cal. | **50** | 3.584 | 3.58203 | 3.46463 | 3.54297 | Pred. |
| **11** | 3.556 | 3.59375 | 3.60074 | 3.69531 | Cal. | **51** | 3.407 | 3.41406 | 3.40799 | 3.4209 | Cal. |
| **12** | 3.787 | 3.78516 | 3.7767 | 3.74707 | Cal. | **52** | 3.437 | 3.44141 | 3.51041 | 3.42969 | Cal. |
| **13** | 3.693 | 3.76172 | 3.63358 | 3.70117 | Pred. | **53** | 3.451 | 3.44141 | 3.44943 | 3.39355 | Cal. |
| **14** | 3.677 | 3.68359 | 3.68234 | 3.72559 | Pred. | **54** | 3.539 | 3.58984 | 3.54654 | 3.57324 | Cal. |
| **15** | 3.82 | 3.71875 | 3.68832 | 3.70215 | Cal. | **55** | 3.59 | 3.55078 | 3.57187 | 3.55469 | Cal. |
| **16** | 3.832 | 3.71484 | 3.61429 | 3.83691 | Pred. | **56** | 3.306 | 3.30078 | 3.29849 | 3.2959 | Cal. |
| **17** | 3.524 | 3.55469 | 3.53854 | 3.55664 | Cal. | **57** | 3.494 | 3.48828 | 3.50345 | 3.48828 | Cal. |
| **18** | 3.3 | 3.33203 | 3.54044 | 3.54688 | Pred. | **58** | 3.641 | 3.61328 | 3.63785 | 3.62402 | Cal. |
| **19** | 3.46 | 3.48438 | 3.48245 | 3.49219 | Pred. | **59** | 3.505 | 3.48828 | 3.46927 | 3.48926 | Cal. |
| **20** | 3.446 | 3.44922 | 3.43967 | 3.46875 | Cal. | **60** | 3.491 | 3.51172 | 3.48422 | 3.49219 | Cal. |
| **21** | 3.541 | 3.54688 | 3.60788 | 3.54297 | Pred. | **61** | 3.751 | 3.73828 | 3.77646 | 3.77246 | Pred. |
| **22** | 3.417 | 3.40625 | 3.46548 | 3.45996 | Cal. | **62** | 3.595 | 3.57812 | 3.64047 | 3.58203 | Cal. |
| **23** | 3.645 | 3.63672 | 3.62369 | 3.66016 | Pred. | **63** | 3.449 | 3.41797 | 3.49918 | 3.50684 | Pred. |
| **24** | 3.71 | 3.74609 | 3.69211 | 3.70312 | Cal. | **64** | 3.676 | 3.65234 | 3.60092 | 3.69629 | Pred. |
| **25** | 3.67 | 3.64844 | 3.6088 | 3.65234 | Cal. | **65** | 3.652 | 3.69141 | 3.66757 | 3.68359 | Cal. |
| **26** | 3.479 | 3.51953 | 3.48807 | 3.50488 | Cal. | **66** | 3.557 | 3.54688 | 3.57211 | 3.54199 | Cal. |
| **27** | 3.766 | 3.76953 | 3.75351 | 3.76562 | Cal. | **67** | 3.088 | 3.09766 | 3.07462 | 3.08594 | Cal. |
| **28** | 3.822 | 3.82812 | 3.77194 | 3.83008 | Pred. | **68** | 3.163 | 3.16016 | 3.19791 | 3.17285 | Cal. |
| **29** | 3.449 | 3.46875 | 3.55856 | 3.47949 | Cal. | **69** | 3.38 | 3.38672 | 3.43045 | 3.4668 | Cal. |
| **30** | 3.212 | 3.23828 | 3.14609 | 3.21582 | Cal. | **70** | 3.686 | 3.66016 | 3.67331 | 3.60059 | Cal. |
| **31** | 3.212 | 3.21875 | 3.38101 | 3.35059 | Pred. | **71** | 3.497 | 3.44141 | 3.44052 | 3.45801 | Cal. |
| **32** | 3.519 | 3.51562 | 3.5632 | 3.59277 | Cal. | **72** | 3.446 | 3.47266 | 3.52023 | 3.55664 | Cal. |
| **33** | 3.464 | 3.42578 | 3.44626 | 3.45312 | Cal. | **73** | 3.306 | 3.31641 | 3.34738 | 3.28027 | Cal. |
| **34** | 3.59 | 3.60938 | 3.57443 | 3.58984 | Cal. | **74** | 3.55 | 3.5625 | 3.55002 | 3.55469 | Cal. |
| **35** | 3.569 | 3.56641 | 3.49484 | 3.5332 | Cal. | **75** | 3.105 | 3.15625 | 3.23538 | 3.32129 | Pred. |
| **36** | 3.404 | 3.41406 | 3.41898 | 3.42383 | Cal. | **76** | 3.251 | 3.28516 | 3.21066 | 3.26074 | Cal. |
| **37** | 3.415 | 3.41797 | 3.48453 | 3.36914 | Pred. | **77** | 3.49 | 3.48438 | 3.49594 | 3.47754 | Cal. |
| **38** | 3.583 | 3.56641 | 3.50418 | 3.54492 | Pred. | **78** | 3.345 | 3.33594 | 3.34653 | 3.35938 | Pred. |
| **39** | 3.457 | 3.46484 | 3.48203 | 3.43945 | Cal. | **79** | 3.176 | 3.18359 | 3.2518 | 3.33105 | Cal. |
| **40** | 3.226 | 3.17188 | 3.22018 | 3.22656 | Cal. | **80** | 3.328 | 3.29297 | 3.26492 | 3.23145 | Cal. |

**Table S2c.** Obtained results with the proposed method for protein parameter.

| **No** | **Ref.** | **Pred.**  **(M5)** | **Pred.**  **(MP5)** | **Pred.**  **(MP6)** | **Set** | **No** | **Ref.** | **Pred.**  **(M5)** | **Pred.**  **(MP5)** | **Pred.**  **(MP6)** | **Set** |
| --- | --- | --- | --- | --- | --- | --- | --- | --- | --- | --- | --- |
| **1** | 8.746 | 8.59375 | 8.65485 | 8.67566 | Cal. | **41** | 8.266 | 8.15625 | 8.25903 | 8.18347 | Pred. |
| **2** | 8.658 | 8.54688 | 8.66809 | 8.73035 | Cal. | **42** | 8.112 | 8.03125 | 7.99725 | 8.10925 | Pred. |
| **3** | 9.125 | 9.04688 | 9.10767 | 9.24011 | Pred. | **43** | 8.112 | 7.95312 | 8.11255 | 8.13171 | Cal. |
| **4** | 9.389 | 9.42188 | 9.40729 | 9.37878 | Cal. | **44** | 8.277 | 8.10938 | 8.20312 | 8.28699 | Cal. |
| **5** | 8.952 | 8.85938 | 9.047 | 8.97449 | Cal. | **45** | 8.496 | 8.39062 | 8.60028 | 8.5614 | Cal. |
| **6** | 8.728 | 8.78125 | 8.80896 | 8.73328 | Cal. | **46** | 8.445 | 8.51562 | 8.42432 | 8.34851 | Cal. |
| **7** | 9.41 | 9.51562 | 9.38794 | 9.32019 | Cal. | **47** | 8.285 | 8.28125 | 8.28833 | 8.28894 | Cal. |
| **8** | 9.595 | 9.46875 | 9.49011 | 9.50378 | Cal. | **48** | 8.994 | 9.07812 | 9.09814 | 9.19714 | Cal. |
| **9** | 9.445 | 9.54688 | 9.41113 | 9.46472 | Cal. | **49** | 8.286 | 8.35938 | 8.27832 | 8.38855 | Cal. |
| **10** | 9.334 | 9.29688 | 9.28638 | 9.25671 | Cal. | **50** | 8.552 | 8.57812 | 8.54968 | 8.58679 | Pred. |
| **11** | 8.504 | 8.51562 | 8.48474 | 8.71277 | Cal. | **51** | 8.423 | 8.54688 | 8.50415 | 8.6073 | Cal. |
| **12** | 8.737 | 8.78125 | 8.7135 | 8.57507 | Cal. | **52** | 8.467 | 8.60938 | 8.39746 | 8.32605 | Cal. |
| **13** | 8.268 | 7.9375 | 7.85889 | 8.01062 | Pred. | **53** | 8.479 | 8.625 | 8.46631 | 8.43347 | Cal. |
| **14** | 7.788 | 7.89062 | 7.95972 | 7.78503 | Pred. | **54** | 8.432 | 8.48438 | 8.39288 | 8.32898 | Cal. |
| **15** | 8.918 | 8.98438 | 9.03333 | 8.84851 | Cal. | **55** | 8.54 | 8.5 | 8.54962 | 8.6532 | Cal. |
| **16** | 9.018 | 9.34375 | 8.79346 | 8.75183 | Pred. | **56** | 7.873 | 7.90625 | 7.89746 | 7.94226 | Cal. |
| **17** | 9.092 | 9.0625 | 9.24976 | 9.2196 | Cal. | **57** | 9.286 | 9.32812 | 9.23364 | 9.22449 | Cal. |
| **18** | 9.452 | 9.29688 | 9.41541 | 9.2821 | Pred. | **58** | 8.381 | 8.34375 | 8.25159 | 8.26746 | Cal. |
| **19** | 9.333 | 9.3125 | 9.31604 | 9.3866 | Pred. | **59** | 8.225 | 8.20312 | 8.2207 | 8.14246 | Cal. |
| **20** | 8.838 | 8.76562 | 8.90369 | 8.99792 | Cal. | **60** | 8.361 | 8.32812 | 8.27844 | 8.32703 | Cal. |
| **21** | 9.711 | 9.67188 | 9.73596 | 9.81726 | Pred. | **61** | 8.986 | 9.07812 | 9.16278 | 9.02625 | Pred. |
| **22** | 9.694 | 9.57812 | 9.54797 | 9.64343 | Cal. | **62** | 9.313 | 9.26562 | 9.20593 | 9.17273 | Cal. |
| **23** | 8.685 | 8.76562 | 8.75305 | 8.75183 | Pred. | **63** | 8.905 | 9 | 9.03796 | 9.10632 | Pred. |
| **24** | 8.729 | 8.70312 | 8.87256 | 8.84656 | Cal. | **64** | 8.338 | 8.21875 | 8.4093 | 8.35925 | Pred. |
| **25** | 8.842 | 8.78125 | 8.76819 | 8.7948 | Cal. | **65** | 8.571 | 8.57812 | 8.65601 | 8.60828 | Cal. |
| **26** | 9.436 | 9.35938 | 9.33093 | 9.36316 | Cal. | **66** | 9.354 | 9.48438 | 9.44342 | 9.30066 | Cal. |
| **27** | 9.179 | 9.21875 | 9.18103 | 9.2157 | Cal. | **67** | 9.021 | 9 | 9.01086 | 8.95203 | Cal. |
| **28** | 8.823 | 9.01562 | 9.13245 | 8.88757 | Pred. | **68** | 9.382 | 9.32812 | 9.38782 | 9.328 | Cal. |
| **29** | 9.592 | 9.45312 | 9.5553 | 9.60632 | Cal. | **69** | 7.654 | 7.79688 | 7.62793 | 7.62488 | Cal. |
| **30** | 8.23 | 8.35938 | 8.21246 | 8.30652 | Cal. | **70** | 7.908 | 7.82812 | 7.97089 | 7.92859 | Cal. |
| **31** | 8.412 | 8.57812 | 8.41492 | 8.64636 | Pred. | **71** | 8.613 | 8.59375 | 8.66211 | 8.61218 | Cal. |
| **32** | 8.412 | 8.39062 | 8.42126 | 8.43835 | Cal. | **72** | 9.073 | 9.01562 | 9.07068 | 9.1405 | Cal. |
| **33** | 8.282 | 8.23438 | 8.41138 | 8.29675 | Cal. | **73** | 7.759 | 7.73438 | 7.72308 | 7.72058 | Cal. |
| **34** | 8.716 | 8.76562 | 8.67847 | 8.6825 | Cal. | **74** | 8.288 | 8.20312 | 8.25244 | 8.21277 | Cal. |
| **35** | 8.435 | 8.42188 | 8.50623 | 8.60046 | Cal. | **75** | 8.649 | 8.67188 | 9.03589 | 9.01257 | Pred. |
| **36** | 8.147 | 8.14062 | 8.1095 | 8.13855 | Cal. | **76** | 7.876 | 8.01562 | 7.90845 | 7.99304 | Cal. |
| **37** | 8.375 | 8.35938 | 8.5141 | 8.30457 | Pred. | **77** | 8.586 | 8.5625 | 8.57617 | 8.53601 | Cal. |
| **38** | 8.506 | 8.60938 | 8.36273 | 8.42664 | Pred. | **78** | 8.03 | 8.3125 | 7.99719 | 8.19812 | Pred. |
| **39** | 8.499 | 8.48438 | 8.41956 | 8.35144 | Cal. | **79** | 8.132 | 8.17188 | 8.15002 | 8.15027 | Cal. |
| **40** | 8.271 | 8.29688 | 8.29419 | 8.2948 | Cal. | **80** | 8.428 | 8.39062 | 8.3999 | 8.35828 | Cal. |

**Table S2d.** Obtained results with the proposed method for starch parameter.

| **No** | **Ref.** | **Pred.**  **(M5)** | **Pred.**  **(MP5)** | **Pred.**  **(MP6)** | **Set** | **No** | **Ref.** | **Pred.**  **(M5)** | **Pred.**  **(MP5)** | **Pred.**  **(MP6)** | **Set** |
| --- | --- | --- | --- | --- | --- | --- | --- | --- | --- | --- | --- |
| **1** | 64.838 | 64.8438 | 64.6891 | 64.8174 | Cal. | **41** | 65.193 | 65.3438 | 65.3209 | 65.0127 | Pred. |
| **2** | 64.851 | 65.2188 | 64.7096 | 64.7002 | Cal. | **42** | 65.217 | 65.0469 | 65.5138 | 65.9717 | Pred. |
| **3** | 63.567 | 63.8906 | 64.0094 | 63.5361 | Pred. | **43** | 65.115 | 65.3281 | 65.1856 | 65.0596 | Cal. |
| **4** | 63.263 | 63.2812 | 63.0504 | 63.5459 | Cal. | **44** | 65.441 | 65.5156 | 65.3062 | 65.3174 | Cal. |
| **5** | 64.148 | 64.3594 | 64.4137 | 64.6064 | Cal. | **45** | 64.67 | 64.8594 | 64.484 | 64.4424 | Cal. |
| **6** | 64.287 | 64.1406 | 64.2516 | 64.4346 | Cal. | **46** | 65.519 | 65.4531 | 65.5924 | 65.5986 | Cal. |
| **7** | 63.513 | 63.3438 | 63.6148 | 63.749 | Cal. | **47** | 65.795 | 65.8281 | 65.7047 | 65.5986 | Cal. |
| **8** | 63.631 | 63.625 | 63.4605 | 63.6611 | Cal. | **48** | 64.253 | 64.2031 | 64.3248 | 64.2939 | Cal. |
| **9** | 63.021 | 62.7656 | 63.5435 | 63.335 | Cal. | **49** | 65.646 | 65.3281 | 65.5201 | 65.374 | Cal. |
| **10** | 63.356 | 63.3594 | 63.4703 | 63.2314 | Cal. | **50** | 64.632 | 64.5625 | 64.4625 | 64.8057 | Pred. |
| **11** | 66.472 | 66.2656 | 66.2848 | 65.5225 | Cal. | **51** | 64.915 | 64.9375 | 64.7887 | 64.3701 | Cal. |
| **12** | 65.386 | 65.4375 | 65.8111 | 65.9482 | Cal. | **52** | 64.882 | 64.8438 | 65.0543 | 65.0186 | Cal. |
| **13** | 65.72 | 65.9531 | 66.0582 | 65.4502 | Pred. | **53** | 64.933 | 64.8438 | 64.8639 | 65.1475 | Cal. |
| **14** | 65.808 | 66 | 65.4635 | 65.9971 | Pred. | **54** | 64.806 | 64.8906 | 64.6847 | 64.9092 | Cal. |
| **15** | 64.544 | 64.7344 | 64.4674 | 64.7939 | Cal. | **55** | 64.757 | 64.7812 | 64.8609 | 64.71 | Cal. |
| **16** | 64.62 | 64.6094 | 65.11 | 64.9854 | Pred. | **56** | 65.337 | 65.4062 | 65.4185 | 65.2275 | Cal. |
| **17** | 63.823 | 64.0156 | 63.6373 | 63.8975 | Cal. | **57** | 64.266 | 64.2656 | 64.0094 | 63.7725 | Cal. |
| **18** | 63.913 | 64 | 63.8033 | 63.8174 | Pred. | **58** | 65.841 | 65.6875 | 65.7232 | 65.8252 | Cal. |
| **19** | 62.826 | 63.0156 | 63.7652 | 63.7666 | Pred. | **59** | 65.374 | 65.3906 | 65.4596 | 65.8584 | Cal. |
| **20** | 64.131 | 64.4219 | 64.2896 | 64.1494 | Cal. | **60** | 65.294 | 65.4219 | 65.3053 | 65.0713 | Cal. |
| **21** | 63.099 | 63.2031 | 63.066 | 62.9482 | Pred. | **61** | 64.539 | 64.5625 | 64.315 | 63.999 | Pred. |
| **22** | 63.246 | 63.6094 | 63.2076 | 63.3506 | Cal. | **62** | 64.367 | 64.3594 | 64.4703 | 64.1846 | Cal. |
| **23** | 65.474 | 65.3594 | 65.3102 | 65.1318 | Pred. | **63** | 64.574 | 64.4844 | 64.818 | 64.2295 | Pred. |
| **24** | 65.427 | 65.5156 | 65.2096 | 65.0283 | Cal. | **64** | 65.547 | 65.4531 | 65.1734 | 65.1709 | Pred. |
| **25** | 64.582 | 65 | 64.648 | 64.8779 | Cal. | **65** | 65.205 | 65.2344 | 65.0523 | 64.958 | Cal. |
| **26** | 63.466 | 63.5312 | 63.7027 | 63.6279 | Cal. | **66** | 63.247 | 63.0781 | 63.1646 | 63.1768 | Cal. |
| **27** | 65.107 | 65.1875 | 64.9986 | 65.0693 | Cal. | **67** | 63.784 | 63.6406 | 63.6627 | 64.0049 | Cal. |
| **28** | 65.601 | 65.2969 | 65.0416 | 65.5791 | Pred. | **68** | 62.884 | 63.125 | 62.8687 | 62.9639 | Cal. |
| **29** | 63.416 | 63.5781 | 63.5416 | 63.5576 | Cal. | **69** | 65.903 | 65.7031 | 65.9742 | 66.1904 | Cal. |
| **30** | 64.261 | 64.25 | 64.4117 | 64.2061 | Cal. | **70** | 65.437 | 65.8594 | 65.3463 | 65.4951 | Cal. |
| **31** | 64.42 | 64.2344 | 64.6676 | 64.6104 | Pred. | **71** | 64.506 | 64.7031 | 64.5094 | 64.54 | Cal. |
| **32** | 64.613 | 64.7188 | 64.5777 | 64.624 | Cal. | **72** | 64.292 | 64.3906 | 64.2291 | 64.0713 | Cal. |
| **33** | 65.104 | 65.0469 | 65.0445 | 65.1514 | Cal. | **73** | 65.524 | 65.4844 | 65.7779 | 65.8271 | Cal. |
| **34** | 64.923 | 64.7656 | 64.9928 | 64.9072 | Cal. | **74** | 64.464 | 64.2969 | 64.5455 | 64.6318 | Cal. |
| **35** | 65.637 | 65.6094 | 65.4244 | 65.1807 | Cal. | **75** | 65.371 | 65.9062 | 65.0221 | 65.1787 | Pred. |
| **36** | 65.022 | 65.25 | 64.9439 | 65.1025 | Cal. | **76** | 65.233 | 65.2344 | 65.2711 | 65.2041 | Cal. |
| **37** | 64.546 | 64.2344 | 64.1734 | 64.9014 | Pred. | **77** | 65.679 | 65.7188 | 65.7164 | 65.8701 | Cal. |
| **38** | 64.787 | 64.9375 | 65.0992 | 64.4893 | Pred. | **78** | 65.144 | 65.2188 | 65.3385 | 65.0986 | Pred. |
| **39** | 64.656 | 64.3906 | 64.8746 | 64.8486 | Cal. | **79** | 65.209 | 65.3281 | 65.0543 | 65.1631 | Cal. |
| **40** | 64.895 | 64.9531 | 64.8531 | 64.7217 | Cal. | **80** | 64.853 | 64.6406 | 65.0133 | 64.8955 | Cal. |

| 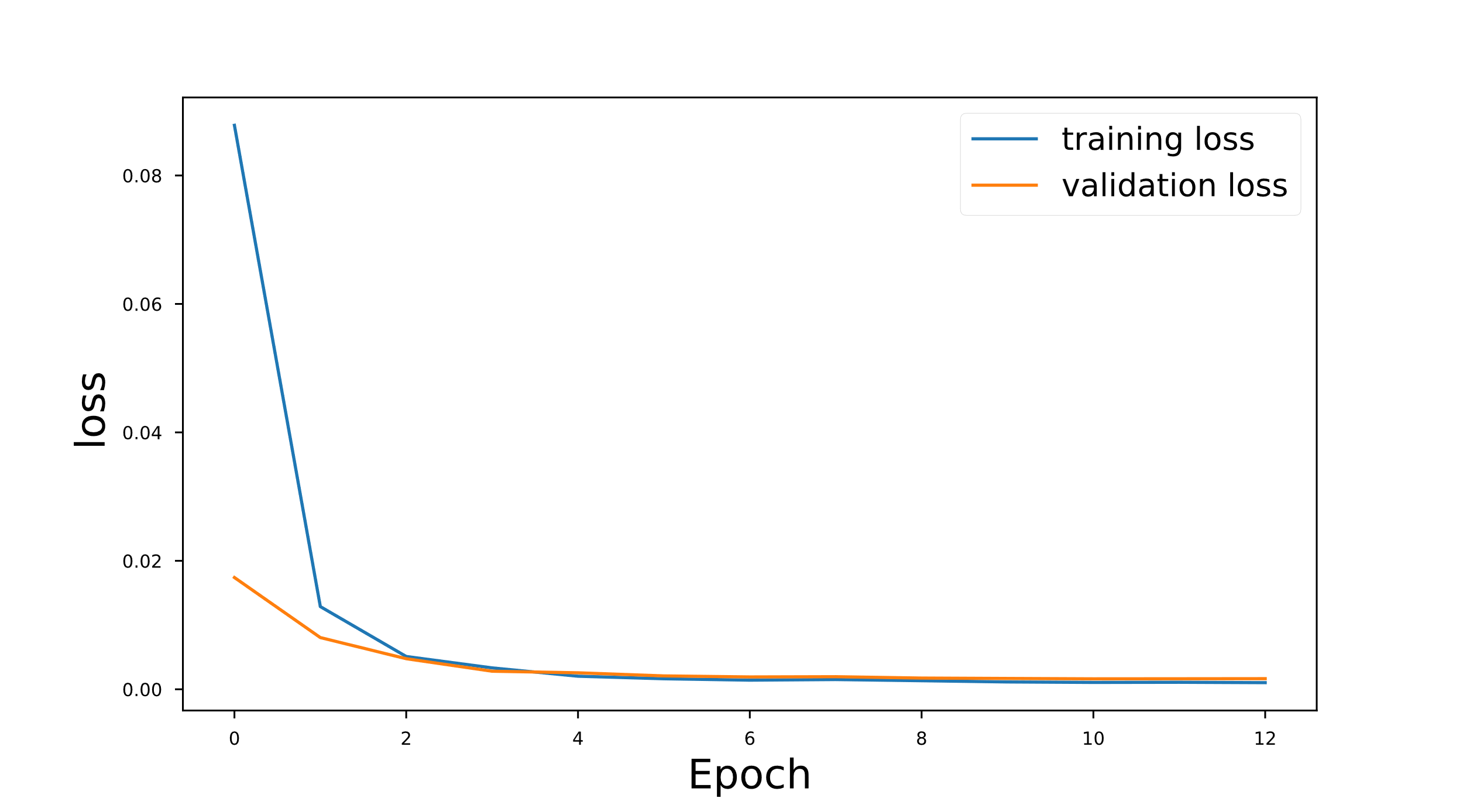 |
| --- |
| a) |
| 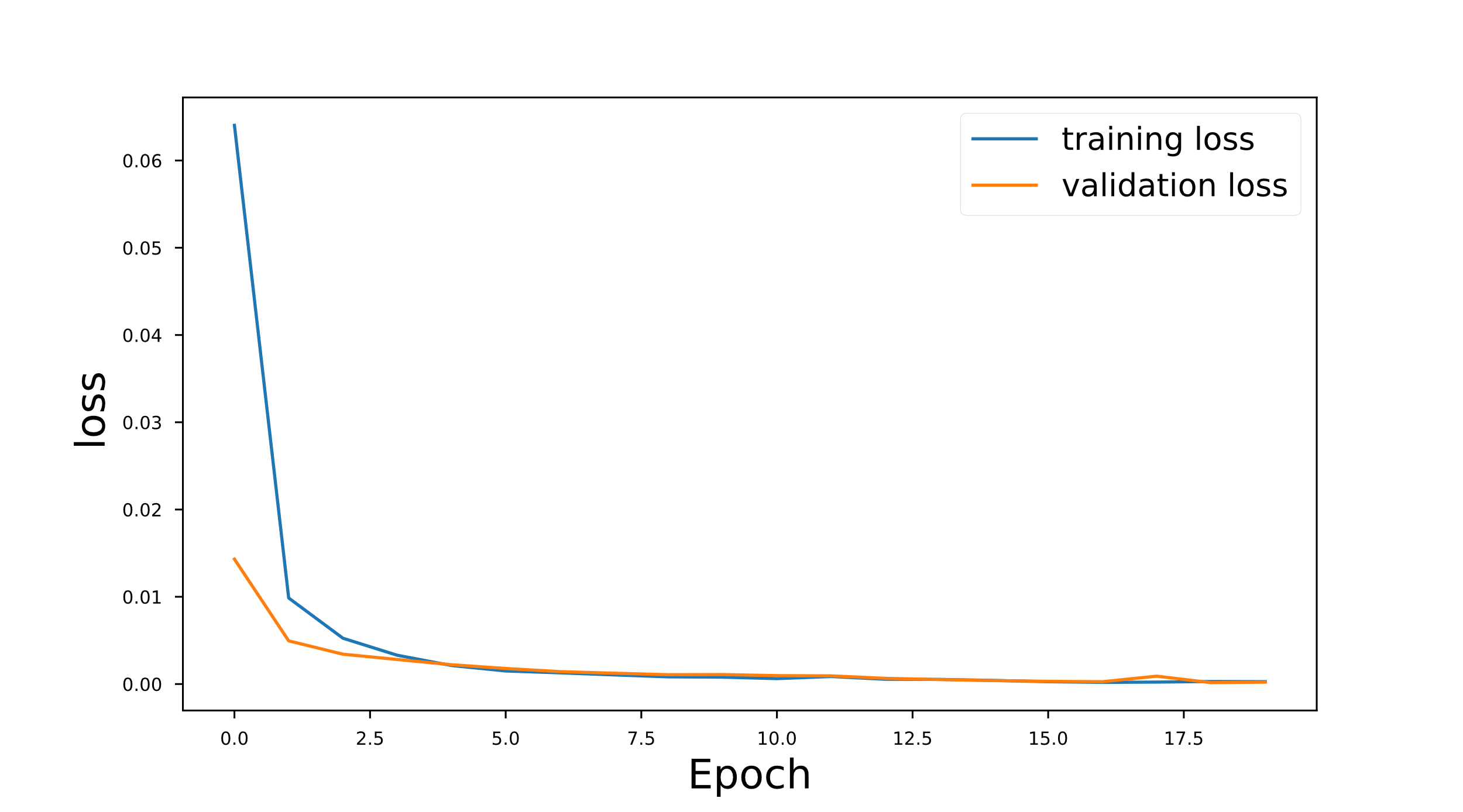 |
| b) |
| 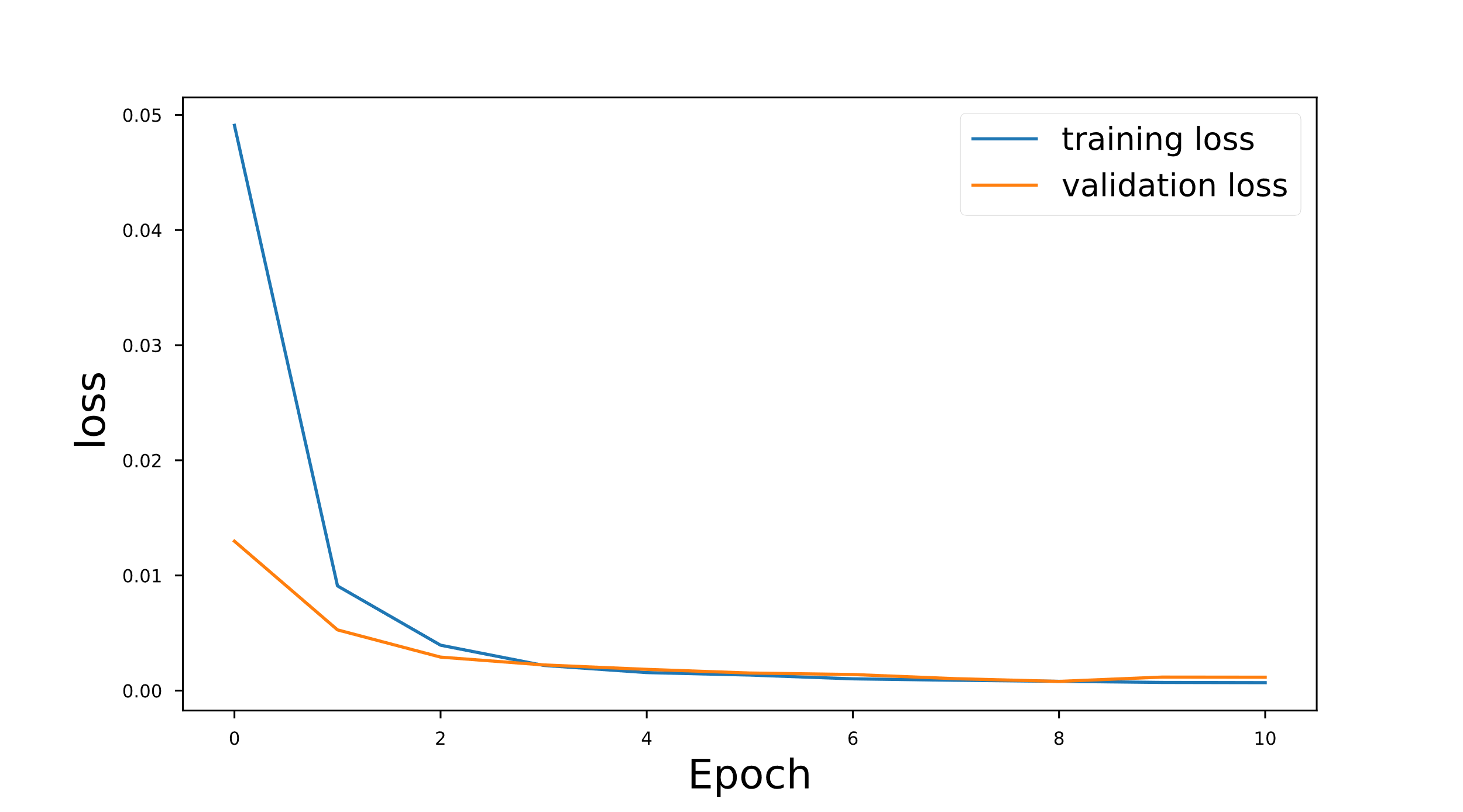 |
| c) |

**Figure S7.** Training and validation loss for a) M5, b) MP5, c) MP6 dataset.
